# Common Data Elements: Critical Assessment of Harmonization between Current Multi-Center Traumatic Brain Injury Studies

**DOI:** 10.1089/neu.2019.6867

**Published:** 2020-05-21

**Authors:** Sacha Meeuws, John K. Yue, Jilske A. Huijben, Nandesh Nair, Hester F. Lingsma, Michael J. Bell, Geoffrey T. Manley, Andrew I. R. Maas

**Affiliations:** ^1^ Department of Neurological Surgery, Antwerp University Hospital and University of Antwerp, Edegem, Belgium.; ^2^ Department of Neurological Surgery, University of California, San Francisco, San Francisco, California, USA.; ^3^ Department of Public Health, Erasmus MC University Medical Center Rotterdam, Rotterdam, the Netherlands.; ^4^ Division of Critical Care Medicine, Children's National Medical Center, Washington, DC, USA.

**Keywords:** clinical trial, Common Data Elements, data standards, InTBIR, standardization, traumatic brain injury

## Abstract

Standardization and harmonization of data collection in studies on traumatic brain injury (TBI) is of paramount importance for meta-analyses across studies. Nearly 10 years ago, the first set of Common Data Elements for TBI (TBI-CDEs v1) were introduced to achieve these goals. The TBI-CDEs version 2 were developed in 2012 to broaden the approach to all ages, injury severity, and phases of recovery. We aimed to quantify the degree of harmonization of these data elements in three large, prospective multi-center studies conducted within the International Initiative for TBI Research (InTBIR). Data variables of the Collaborative European NeuroTrauma Effectiveness Research in Traumatic Brain Injury (CENTER-TBI; adult and pediatric patients in Europe and Israel), Transforming Research and Clinical Knowledge in Traumatic Brain Injury (TRACK-TBI; adult and pediatric patients in the U.S.), and Approaches and Decisions in Acute Pediatric TBI (ADAPT; international study on severe pediatric TBI) studies were indexed and matched to the second version of the TBI CDEs. We focused on the CDE sub-categories of “Acute Hospitalized” (AH) and “Moderate/Severe TBI: Rehabilitation (Rehab). All “Core” and “Basic” level CDEs were considered. Closely related elements were reduced to one variable to prevent over-representation. Categorical elements and text elements for the same variable were likewise merged to one element for analysis. Following reduction and merging of related elements, 21 Core, 46 Basic AH, and 50 Basic Rehab elements were deemed harmonizable across studies. Gaps in global applicability were identified for four of the TBI CDEs and many of the outcome instruments, which are only available in the English language. Agreements of Core and Basic study CDEs for the AH domain with the TBI CDEs were respectively 81% and 91% for CENTER-TBI, 76% and 93% for TRACK-TBI, and 85% in ADAPT for both domains. For the domain Rehab, agreement with Basic TBI CDEs was 84% for CENTER-TBI, 94% for TRACK-TBI, and 71% for ADAPT. Non-harmonization was largely caused by absence of the elements in the studies. For elements present, the compatibility of coding with TBI CDEs was 90-99%. The degree of harmonization was greatest between CENTER-TBI and TRACK-TBI with 81-87% overlap within the TBI CDE sub-categories. The high degree of harmonization of study variables among these studies demonstrates the importance and utility of common data elements in TBI research. It also confirms the potential for future meta-analyses across these large studies, especially for CENTER TBI and TRACK TBI. The global applicability of the TBI CDEs needs to be improved for them to become a global standard for TBI research. CENTER-TBI, TRACK-TBI, and ADAPT, along with other studies within the InTBIR Initiative, provide a platform to inform further refinement and internationalization for the next version of the TBI CDEs.

## Introduction

Traumatic brain injury (TBI) poses a huge global public health problem. A critical need exists for robust clinical research on TBI, involving large scale studies, multi-center international collaborations and data sharing.^1^ Funding agencies are strongly calling for data sharing between and meta-analyses across studies. This requires a “common language” for data collection, in terms of what variables to record and how to code them.

The development of uniform data standards—termed “common data elements (CDEs)”—was initiated by the International Mission for Prognosis and Analysis of Clinical Trials in TBI (IMPACT) study group^2,3^ and taken forward by an international group of 149 institutes and agencies supported, among others, by the United States National Institute of Neurological Disorders and Stroke (NINDS), U.S. Department of Defense, U.S. Department of Education, and the U.S. Department of Veteran's Affairs. This consensus effort lead to version 1 of the TBI CDEs (TBI-CDE v1), published in 2010.^4^ In 2012, a re-structuring was introduced with the overarching aim of creating a set of “Core” CDE elements suitable for use in all TBI studies.^5^ TBI is arguably the most heterogeneous of all neurological disorders, which makes the standardization effort really challenging, but also indicates a need for flexibility and raises the question if defining subgroups within the CDEs might be desirable.

For these reasons, sets of “Basic” elements were introduced with the following four sub-categories of clinical TBI studies.: “Concussion/Mild TBI”; Acute Hospitalized (AH)”; “Moderate/Severe TBI: Rehabilitation (Rehab)”; and “Epidemiology.” A larger set of “Supplemental” elements was created to allow flexibility in adapting to unique study criteria and end-points. This second version, TBI-CDE v2, is hosted and maintained by NINDS (www.commondataelements.ninds.nih.gov).

Since 2012, the TBI-CDEs have undergone several updates based on input from expert working groups, researchers and funding agencies. We explored the degree of harmonization across three large studies of TBI, conducted under the umbrella of the International Initiative for TBI Research (InTBIR: https://intbir.nih.gov),^6^ using the TBI-CDE v2 and discuss the evolution of the CDEs in the context of achieving global applicability to support data sharing and international collaboration.

## Methods

### Included studies

Data elements of Case Report Forms (CRFs) and imaging data repositories were extracted from three large multi-center observational studies conducted under the umbrella of InTBIR:

- Collaborative European NeuroTrauma Effectiveness Research in Traumatic Brain Injury (CENTER-TBI: www.center-tbi.eu).^7,8^ CENTER-TBI recruited and analyzed 4509 patients (4254 adults and 255 pediatric) with TBI of all severities in Europe and Israel. The analysis is in two directions: Improved characterization in the context of developing precision medicine approaches, and identification of best practices using a comparative effectiveness design.
- Transforming Research and Clinical Knowledge in Traumatic Brain Injury (TRACK-TBI: https://tracktbi.ucsf.edu).^9^ TRACK-TBI recruited 2698 patients (2553 adults, 145 pediatric) with TBI of all severities in the U.S. and 299 orthopedic trauma controls. The main focus is on improved characterization of TBI to inform precision medicine (biomarkers, classification, prognosis, systems of care, management, and interventions) approaches to both research and clinical management.
- Approaches and Decisions in Acute Pediatric Traumatic Brain Injury (ADAPT: www.adapttrial.org).^10^ ADAPT recruited 1000 pediatric patients with severe TBI across five continents. The main aims are identification of best practices for six first-tier therapies for intracranial hypertension and basic clinical care using statistical approaches commonly employed in comparative effectiveness research.

### TBI CDEs and indexing process

We extracted TBI-CDE v2 from the NINDS Common Data Elements Web site (https://commondataelements.ninds.nih.gov) on August 20, 2015, which was close to the times of initiation of the three studies. We focused on the domains “Acute Hospitalized (AH)” and “Moderate/Severe TBI: Rehabilitation (Rehab),” as these were considered most relevant to the three studies. “Core” and “Basic” level CDEs were extracted into an Excel overview and listed by respective TBI CDE v2 identification codes. An overview of all Core and Basic CDEs for the AH and Rehab domains and CRF modules can be found on the Data Standards tab of the NINDS CDE Traumatic Brain Injury Web site. General Core CDEs are defined as data elements considered mandatory for all NINDS funded studies on neurological diseases (e.g., epilepsy, stroke). Disease Core CDEs are required data elements for disease specific studies, such as traumatic brain injury. Basic CDEs are defined as elements that should be included in studies related to the section of interest. “Supplemental” level CDEs were not included in the current analysis.

Many Core and Basic CDEs required re-formatting in preparation of analyses for this study. Various elements could relate to a single variable. For example, the Craig Handicap Assessment Reporting Technique Short Form (CHART-SF), an outcome instrument, was represented by a total of 29 CDEs. All such closely related elements were reduced to one variable in order to prevent over-representation and allow fair comparison. Variables that consisted of separate categorical and text elements were likewise merged to one element for analysis. In the approach we undertook, driving factors for considering an element “harmonizable” were “global applicability” and intended use in a general setting. Consequently, elements that were not globally applicable (for example Race and Ethnicity—USA category) were excluded, as were elements solely applicable to specific sub-populations, such as the pediatric population or military setting. Pediatric elements were retained for analysis of ADAPT and pediatric versions of the Glasgow Coma Scale (GCS) and Glasgow Outcome Scale (GOS) were considered compatible with the adult versions. The CDE indexing process for the current analysis was performed by two coauthors (S.M.; J.K.Y.). When consensus was equivocal, the senior author (A.I.R.M.) was queried for adjudication.

### Data extraction and analysis

Data elements from the e-CRFs of the three studies were indexed and matched in an Excel (Supplementary File S1) to the TBI-CDEs v2 (http://www.commondataelements.ninds.nih.gov). For TRACK-TBI and ADAPT, imaging elements were derived from the imaging repositories, as these had been scored separately at central review and no results of imaging studies directly entered by site study staff in the e-CRF. We restricted our analysis of imaging elements to those listed in AH and Rehab domains of the NINDS CDEs.

The TBI-CDE v2 retained following the reduction and exclusion processes described above were considered “harmonizable.” Each CDE for the three studies was scored dichotomously (yes/no) for its presence and compatibility with TBI-CDE v2 codings. Compatibility was defined as either an identical coding format, or a coding format that included the essential elements of coding found on the NINDS Web site. We calculated both the number and percentage of CDEs present in each study and their compatibility with the TBI-CDE v2, and the harmonization of data elements between studies were calculated. Sensitivity analysis was performed on basic CDEs common or unique to the AH and Rehab domains, as many of these basic elements overlapped. Descriptive statistics were used to present data in tabular formats.

## Results

### TBI CDEs

The NINDS subsection “Acute Hospitalized” consisted of 134 CDEs (29 “Core,” 105 “Basic”), and the Rehab subsection contained 162 CDEs (29 “Core,” 143 “Basic”). Core variables were identical for the AH and Rehab domains. Of the 29 Core elements, seven were General, and 22 TBI-specific. A large overlap (*n* = 48) was noted between the Basic elements of the AH and Rehab domains. Twelve elements were unique to the AH domain and 15 to the Rehab domain (Supplementary Table S1).

**FIG. 1.**
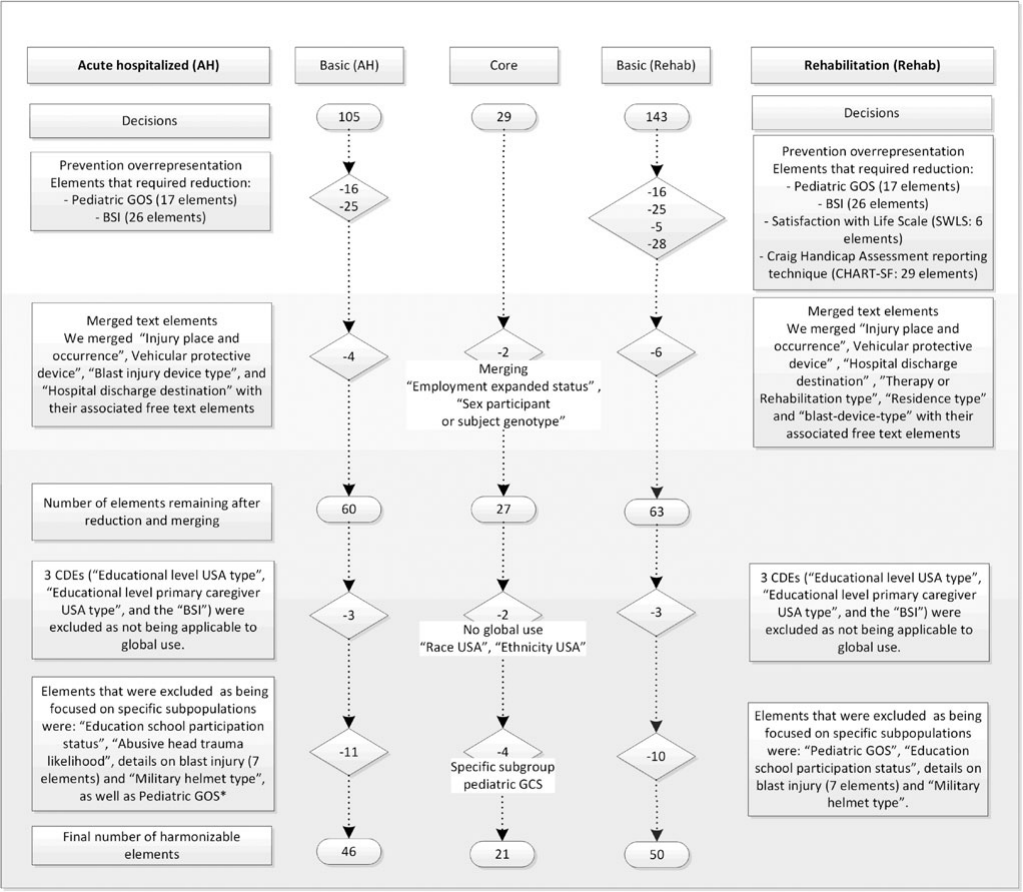
Flowchart to illustrate decision-making process, resulting in harmonizable Common Data Elements. The National Institute of Neurological Disorders and Stroke Common Data Elements (CDEs) list 142 elements for the basic” Rehab” domain. However, element C05400 (Injury date time) is likely misclassified as Core element. Elements that were added for harmonization for the Approaches and Decisions in Acute Pediatric Traumatic Brain Injury (ADAPT) study were “educational level USA type,” “educational level primary caregiver USA type,” “education school participation status,” “abusive head trauma likelihood” for the Acute Hospitalization domain, and “educational level USA type,” “educational level primary caregiver USA type,” “education school participation status” for the rehabilitation domain. *For comparison of ADAPT to NIH CDEs and to CENTER-TBI and TRACK-TBI, we considered the pediatric versions of Glasgow Coma Scale and Glasgow Outcome Scale-Extended as identical to the adult versions. ^#^The Brief Symptom Inventory was considered not globally applicable as it is only available in the English language and is copyrighted, precluding general use.

Basic elements that required reduction in order to prevent over-representation in the AH domain included the pediatric Glasgow Outcome Scale (GOS; 17 elements) and the Brief Symptom Inventory (BSI; 26 elements); and in the Rehab domain, Pediatric GOS (17 elements, BSI (26 elements), Satisfaction with Life Scale (SWLS; six elements), and CHART-SF (29 elements). Reduction and merging of elements with their related free text elements reduced the number to 27 Core, 60 Basic AH, and 63 Basic Rehab elements (Fig. 1). Of these, two General Core elements (“Race USA category” and “Ethnicity USA category”) and three Basic CDEs (“educational level USA”, “educational level primary caregiver USA type,” and the BSI) in both the AH and Rehab domains were excluded as not being applicable to global use. The BSI, a copyrighted instrument, was excluded as it is not freely available, being copyrighted, and because it is only available in the English language. A total of 15 CDEs (four Core and 11 basic) were excluded as they targeted specific sub-populations (pediatric and military).

Pediatric elements were retained for comparison of ADAPT (three Basic AH and two Basic Rehab), and elements not relevant to the pediatric population of severe TBI in ADAPT were excluded (Supplementary Table S2). A total of 21/27 (78%) “Core,” 46/60 (77%) “Basic” in the AH domain, and 50/63 (79%) “Basic” in the Rehab domain were considered “harmonizable” with the reduced NINDS CDE v2 in the general adult population (Fig. 1). For the pediatric population of severe TBI in ADAPT, 20 “Core,” 41 “Basic AH,” and 41 ”Basic Rehab” were considered “harmonizable.”

### Harmonization of study CDEs versus TBI-CDE v2

A summary overview of the compatibility of study elements with the “harmonizable” NINDS CDE v2 is presented in Table 1. Presence and compatibility with the NINDS codings ranged from 71 to 94% across studies for the three domains. The degree of harmonization of study CDEs with TBI-CDEs v2 and between studies was mainly determined by non-presence of elements. Higher degrees of harmonization were found in CENTER-TBI and ADAPT for the CDEs of the AH domain compared with the Rehab domain. Harmonization for TRACK-TBI was 94% for both domains. Sensitivity analysis showed substantially lower harmonization of CDEs unique to the Rehab domain in CENTER-TBI and ADAPT compared with CDEs common to AH and Rehab domains and to CDEs unique to the AH domain (Table 2). For unique elements present across the Core, Basic AH, and Basic Rehab domains, the compatibility with the NINDS codings ranged from 97 to 99% (CENTER-TBI, 69/71 (97%); TRACK-TBI, 73/74 (99%); and ADAPT, 56/58 (97%; Supplementary Table S3).

**Table 1.** Presence and Compatibility of Study Elements with CDEs

Studies	Core* n/N *(%)	Basic AH* n/N *(%)	Basic Rehab* n/N *(%)
CENTER-TBI	17/21 (81%)	42/46 (91%)	42/50 (84%)
TRACK-TBI	16/21 (76%)	43/46 (93%)	47/50 (94%)
ADAPT	17/20^*^ (85%)	35/41^#^ (85%)	29/41^ (71%)

^*^ Element C18658 Employment Expanded status is not applicable to ADAPT.

# Eight elements are not applicable to ADAPT as they are not relevant to the study population of pediatric patients with severe TBI (see Supplementary Table S2). Three basic “AH” variables that had been excluded from the comparisons for adult studies are relevant to the pediatric population of ADAPT; these concern “education school participation,” “abusive head trauma,” and the “pediatric GOS.” These elements were present in ADAPT.

^Eleven elements are not applicable to ADAPT as they are not relevant to the study population of pediatric patients with severe traumatic brain injury (see Supplementary Table S2). Two basic Rehab variables that had been excluded from the comparisons for adult studies are relevant to the pediatric population of ADAPT; these concern “education school participation” and the “pediatric GOS.” These elements were present in ADAPT.

AH, Acute Hospitalized; CENTER-TBI, Collaborative European NeuroTrauma Effectiveness Research in Traumatic Brain Injury; TRACK-TBI, Transforming Research and Clinical Knowledge in Traumatic Brain Injury; ADAPT, Approaches and Decisions in Acute Pediatric Traumatic Brain Injury; GOS, Glasgow Outcome Scale.

**Table 2.** Sensitivity Analysis of Basic Elements

Studies	Basic Elements Common to AH and Rehab* n/N *(%)	AH Unique n/N (%)	Rehab Unique* n/N *(%)
CENTER-TBI	32/35 (91%)	10/11 (91%)	10/15 (67%)
TRACK-TBI	33/35 (94%)	10/11 (91%)	14/15 (93%)
ADAPT	25/29^*^ (86%)	10/12^#^ (83%)	4/12^ (33%)

^*^ Eight common basic elements are not applicable to ADAPT as they are not relevant to the study population of pediatric patients with severe traumatic brain injury (see Supplementary Table S2). Two basic elements common to “AH” and” Rehab” that had been excluded from the comparisons for adult studies are relevant to the pediatric population of ADAPT; these concern “education school participation” and the “pediatric GOS.” These elements were present in ADAPT.

# One basic element unique to AH that had been excluded from the comparisons for adult studies is relevant to the pediatric population of ADAPT: “abusive head trauma.”

^Three unique Rehab elements are not applicable to ADAPT as they are not relevant to the study population of pediatric patients with severe traumatic brain injury (see Supplementary Table S2). These concern “marital status,” “SWLS,” and the “CHART-SF.”

AH, acute hospitalized; CENTER-TBI, Collaborative European NeuroTrauma Effectiveness Research in Traumatic Brain Injury; TRACK-TBI, Transforming Research and Clinical Knowledge in Traumatic Brain Injury; ADAPT, Approaches and Decisions in Acute Pediatric Traumatic Brain Injury.

### CENTER-TBI

Of the five harmonizable CDEs in the category “General (For all diseases)” with classification “Core,” only three were present in the CENTER-TBI data. The elements “birth date” and “medical history condition Snomed [Systematized Nomenclature of Medicine] CT code” were not present. In the European Union, birth date is considered a potential patient identifier and was thus excluded from the data collection. CENTER-TBI did record “age,” which is listed as a separate basic element in the AH section. The compatibility of the CENTER-TBI CDEs with the section “General” was thus only 60% compared with 88% (14/16) for the TBI specific Core CDEs. Of all “Core” CDEs (general and TBI specific), a total of 18 were present, of which 17 were compatible (Table 1). Coding of cause of injury was not compatible with the TBI-CDE v2 “Injury ICD external cause.” In the AH section, 42 basic elements were present and compatible. Details of non-present and non-compatible elements are summarized in Supplementary Tables S3A, S3B, S3C. Overall, 81% of the harmonizable Core and 91% of the harmonizable Basic AH elements were present and compatible.

In the sub-disease “Moderate/Severe TBI: Rehabilitation” section, a total of 42 “Basic” CDEs were “present” and “compatible,” corresponding to a harmonization rate of 84%.

### TRACK-TBI

Of the five harmonizable CDEs in the category “General (for all diseases)” with classification “Core,” three were present in the TRACK-TBI data and compatible with TBI-CDE v2 coding. The elements “birth date” and “medical history condition Snomed CT code” were not present. Like CENTER-TBI, TRACK-TBI did record “age,” which is listed as a separate basic element in the Acute Hospitalized section. “Birth date” was collected locally by each site, kept confidential and secure, but not stored in the database. Of all the “Core” CDEs, 16 were present and “compatible.” This corresponds to a compatibility of 76% with the harmonizable Core TBI-CDEs v2 (Table 1). In the sub-disease section AH, 43 Basic elements were “present” and “compatible,” corresponding to a harmonization rate of 93%. In the sub-disease “Moderate/Severe TBI: Rehabilitation” section, 47 out of 50 (94%) harmonizable elements were present and compatible. Details of non-present and non-compatible elements are summarized in Supplementary Tables S3A, S3B, S3C.

### ADAPT

Of the five harmonizable CDEs in the category “General (For all diseases)” with classification “Core,” two (“Birth date” and “Gender”) were present in the ADAPT data and compatible with NINDS coding. In addition, the non-global elements “Ethnicity USA category” and “Race USA Category” were present. A total of 17 “Core” CDEs were present and compatible (Table 1). One Core element (“Employment Expanded status”) was considered not relevant to the pediatric population. The Glasgow Outcome Scale-Extended (GOSE) was replaced by the pediatric version. The overall compatibility of core elements was 17/20 (85%).

In the sub-disease “Acute Hospitalized” section,” a total of 35 basic elements were considered present and compatible out of 41 harmonizable basic elements relevant to ADAPT's specific population of severely injured pediatric patients. This corresponds to a compatibility of 85% for basic elements. Three basic AH variables (“education school participation,” “abusive head trauma,” and the “pediatric GOS”) excluded from comparisons with the adult studies were considered relevant to the pediatric population of ADAPT and included in the matching. These elements were present in ADAPT. Basic CDEs not present or not compatible are summarized in Supplementary Tables S3A, S3B, S3C. The elements “military deployment indicator” and elements (*n* = 7) related to loss of consciousness, alteration of consciousness, and post-traumatic amnesia were not applicable to ADAPT's study population. Similarly, the Marshall CT classification was not considered appropriate for the pediatric population by the ADAPT Investigators.

In the sub-disease “Moderate/Severe TBI: Rehabilitation” section, a total of 29 “Basic” elements of 41 harmonizable CDEs relevant to the study population of ADAPT were “present” and “compatible” (71%).

### Harmonization across studies

The degree of harmonization across studies is presented in Table 3. For the Core and AH domains, the harmonization ranged from 75% to 87% and for the Rehab domain from 64% to 82%. For each domain the degree of harmonization was greatest between CENTER-TBI and TRACK-TBI. Non-harmonization was largely caused by absence of a CDE (Supplementary Table S2) in one or both of the studies being compared. Sensitivity analysis showed poor harmonization for Unique elements of the Rehab domain between the adult studies (CENTER- and TRACK-TBI) and ADAPT (Table 4).

**Table 3.** Study to Study Comparisons

Studies	Core* n/N *(%)	Basic AH* n/N *(%)	Basic Rehab* n/N *(%)
CENTER-TRACK	17/21 (81%)	40/46 (87%)	41/50 (82%)
CENTER-ADAPT	16/20 (80%)	31/38 (82%)	25/39 (64%)
TRACK-ADAPT	15/20 (75%)	33/38 (87%)	28/39 (72%)

*n/N* (%): n harmonized/*N* harmonizable.

For Core, one element is not relevant to ADAPT: (C18658 Employment expanded status). For AH, Eight elements are not applicable to ADAPT (see Supplementary Table S2) as they are not relevant to the study population of pediatric patients with severe traumatic brain injury. For Rehab, 11 elements are not applicable to ADAPT (see Supplementary Table S2) as they are not relevant to the study population of pediatric patients with severe traumatic brain injury.

AH, Acute Hospitalized; CENTER-TBI, Collaborative European NeuroTrauma Effectiveness Research in Traumatic Brain Injury; TRACK-TBI, Transforming Research and Clinical Knowledge in Traumatic Brain Injury; ADAPT, Approaches and Decisions in Acute Pediatric Traumatic Brain Injury.

**Table 4.** Study to Study Comparisons: Sensitivity Analysis of Basic Elements

Studies	Basic Elements Common to AH and Rehab* n/N *(%)	AH Unique* n/N *(%)	Rehab Unique* n/N *(%)
CENTER-TRACK	31/35 (89%)	10/11 (91%)	10/15 (67%)
CENTER-ADAPT	23/27 (85%)	8/11 (73%)	2/12 (17%)
TRACK-ADAPT	24/27 (89%	9/11 (82%)	4/12 (33%)

*n/N* (%): *n* harmonized*/N* harmonizable.

Eight basic elements common to “AH” and “Rehab” are not applicable to ADAPT (see Supplementary Table S2) as they are not relevant to the study population of pediatric patients with severe TBI. Three elements unique to Rehab are not applicable to ADAPT (see Supplementary Table S2) as they are not relevant to the study population of pediatric patients with severe TBI. These concern “marital status,” “SWLS,” and the “CHART-SF.”

AH, Acute Hospitalized; CENTER-TBI, Collaborative European NeuroTrauma Effectiveness Research in Traumatic Brain Injury; TRACK-TBI, Transforming Research and Clinical Knowledge in Traumatic Brain Injury; ADAPT, Approaches and Decisions in Acute Pediatric Traumatic Brain Injury; SWLS, Satisfaction with Life Scale; CHART-SF, Craig Handicap Assessment Reporting Technique Short Form.

## Discussion

In this study we aimed to explore how the common data elements proposed for studies of TBI as of 2012, the TBI-CDEs v2, have been implemented into three of the larger InTBIR studies, and to quantify the degree of harmonization across studies. To our knowledge, this is the “first in its kind” study to systematically evaluate the implementation and harmonization of TBI-CDEs across large scale studies. We found good harmonization for Core and Basic AH CDEs for all studies (76-93%), but lower rates for basic Rehab CDEs in ADAPT compared with the Basic AH CDEs. The harmonization between studies ranged from 64 to 87% for the three domains, and was greatest for CENTER-TBI and TRACK-TBI. This is not surprising as both studies focused on similar populations and were designed collaboratively with plans for future meta-analyses and collaborative studies.

The relatively lower harmonization with ADAPT is understandable as this study focused exclusively on severely injured, unconscious children—leading many CDEs to be irrelevant (e.g., -traumatic amnesia assessments, employment history, and others). Incomplete harmonization was largely attributable to the absence of the CDEs from the study design. For CDEs present, the compatibility with recommended CDE codings was high. The overall high degree of compatibility of study elements with the TBI-CDEs v2 and the strong harmonization between studies illustrates a high degree of standardization and confirms the feasibility of meta-analyses across studies. The final demonstration of utility and importance of TBI-CDEs will come from such meta-analyses, which are planned to be conducted following completion of the primary analyses of the studies.

Matching of the study variables to the TBI-CDEs v2 was challenging (Table 5) and revealed issues that could affect the global applicability of the CDEs. The presence of potential patient identifiers, such as birth date, is of concern and should be corrected. We found substantial overlap (75-80%) between the basic CDEs for the AH and Rehab domains, and therefore conducted a sensitivity analysis exploring harmonization for CDEs common to both domains and unique to either AH or Rehab domains.

**Table 5.** Isuues with CDEs

- Listing of Core and Basic CDEs contain duplicates - Multiple elements exist for one variable - Substantial overlap between Basic CDEs for AH and Rehab domains - Inclusion of potential patient identifiers - Discrepancies in classification of Core vs Basic vs Supplemental - Adult and pediatric versions of the same variable included as separate CDEs, sometimes with different classification

CDE, Common Data Elements; AH, Acute Hospitalized.

In some instances, discrepancies were noted in the classification of CDEs as Core, Basic, and Supplemental. These have been brought to the attention of the TBI CDE team at NINDS. The current format of the CDEs includes multiple elements for the same variable. Examples are the outcome instruments (e.g., Pediatric GOS,17 elements; SWLS, six elements; BSI, 26 elements; and CHART-SF, 29 elements). Other variables had separate elements for categorical and free text entries. In order to prevent over-representation of these variable, they were reduced to one element for our matching process. Various inconsistencies were noted in the listing of some variables. For example, the GOSE is listed as one element in the Core section of the CDEs, but the pediatric version listed in the Basic AH section comprised of 17 elements. We recognize that data managers and clinical researchers may have different perspectives on the ontology for listing CDEs. For example, listing of all sub-items of, for example, outcome instruments as separate data elements may be logical from a data management perspective, but results in a long—and perhaps intimidating—list of elements. Whichever approach is chosen, it should be consistent. User-friendliness and oversight should be considered in the presentation of CDEs. The inclusion of a modular presentation format in the current version of the CDEs largely meets these considerations, but the classification of the CDEs (e.g., Core or Basic) is less clear. The recent addition of case report forms for the specific study types (e.g., Acute Hospitalized) also helps to organize the TBI CDEs for data collection.

The presence of sub-population specific elements (e.g., pediatric, military) in the NIH-NINDS CDEs complicated the matching process of study data elements to the CDEs. We decided not to include these elements in the matching of studies primarily focused on the general civilian and mainly adult population, but retained the pediatric variables for matching of ADAPT variables to CDEs. We recognize that excluding subpopulation-specific elements may be debatable. However, it may be more appropriate for such sub-population-specific elements to be accorded special status in a dedicated section.

We found it surprising that the Rehab domain contained more elements with greater detail on pupillary reactivity than the AH section, which appears counter-intuitive. Moreover, the current version of the TBI CDEs, version 2, does not provide recommendations on timing of assessments. For some variables, such as the GCS, it may be relevant to record assessments at multiple time-points, for example pre-hospital, on presentation, and post-resuscitation. Overall, a total of three NINDS CDEs were not prospectively collected in any of the studies (one Core, C17396 “Sex participant or subject genotype”; one basic CDE in both the “AH” and “Rehab” domains, “Death cause ICD-9; 1 specific to the Rehab domain: Therapy Rehab ICD-9 code”). The absence of these CDEs in any of the studies casts doubts on their applicability in TBI research.

### CDEs as global standards

We consider the efforts towards standardization of data collection across studies of paramount importance and strongly advocate the development of global standards. The opportunities to conduct meta-analyses, at least across CENTER-TBI and TRACK-TBI, offer huge opportunities due to the power of larger numbers, and global standards will increase these further. Differences in care and outcome can be explored in comparative effectiveness research (CER), allowing identification of best practices. In CER analysis, multivariable random effects regression models with an instrument on hospital level (hospital policy) are used, adjusted for case-mix to explore relations between different (treatment) policies and outcome. These represent some of the key aims of both CENTER-TBI and TRACK-TBI.

We have identified gaps in the current version of the TBI-CDEs v2 that limit their international use. Several of the elements are U.S.-centric and relate to reporting requirements for NIH funding. These elements, such as race and ethnicity, are collected differently in other countries, if they are collected at all. The reliability of this self-reported information has been questioned and in our current age of genomic research will likely be replaced with more objective ancestry informative markers.^11^ Educational level is an important predictor of outcome in mild TBI, but the current CDE coding is U.S.-centric. We recognize that educational systems vary across the world, and suggest that mapping of different codings should be feasible. Other obstacles on the path toward global standards are that some of the patient-reported outcome measures listed in the TBI-CDEs version 2 are proprietary and copyrighted, and most are only available in the English language. The linguistic validations of many outcome instruments performed by the CENTER-TBI project constitute a major accomplishment towards more global applicability. However, the BSI, SWLS, and CHART-SF, listed as basic elements in the CDEs were not included in these validation efforts. We contend that outcome instruments recommended in the TBI-CDEs v2 should be freely available to the clinical research community. The BSI is copyrighted, and not freely available. The SWLS is copyrighted, but may be used free of charge. Of particular concern is that of the seven Core CDEs considered as “General” (e.g., applicable and required across all neurological diseases), two were not globally applicable and a further two (“Birth date” and “Med. history Snomed”) were not included in either CENTER-TBI or TRACK-TBI due to conflicts with existing privacy legislation.

### Limitations

Several limitations of this harmonization study should be acknowledged:

First, we based the matching process of study data elements to the TBI-CDEs v2 as of Aug 20th, 2015. This was a deliberate choice as our intent was to map the study data to the version of the CDEs current at the time of study initiation. We recognize that since then the TBI-CDEs have evolved and the Web site has been updated. Compared with the 2015 version, eight new Core elements have been added (Supplementary Table S4). However, the coding of existing variables has not changed substantially. The new CDEs concerned questionnaire assessments: The “Test of Everyday Attention for Children (TEACH) and the “Clinician Administered PTSD Scale (CAPS; seven elements)”. Many of these new CDEs contained clear patient identifiers such as “Subject name,” “child name,” or “relative name.” We consider this undesirable and strongly recommend that they be excluded as Core elements in the future.

Second, the matching and critical analysis cannot be considered representative of all CDEs. We focused on the “General” and “AH” CDEs as these were most relevant to the three InTBIR studies included in this report. We did not include the CDE subsections on “epidemiology” or “concussion.” Third, for purposes of analysis, we re-formatted the CDEs by reducing the number of related elements (e.g., outcome instruments) to one, in order to avoid over-representation and excluded variables that were only applicable to specific sub-populations. We considered this a fair and transparent process, but recognize that as a consequence, the percentage of study variables present in the study datasets was higher than if matching had been performed versus the full list of original CDEs. Fourth, while the assessment of whether an element was present or not is objective, the judgment of “compatibility” includes, by definition. a subjective component.

## Conclusion

Our analysis highlights several achievable goals that would powerfully evolve the TBI-CDEs into true trans-global utility. First, we found substantial overlap between Basic CDEs in the domains “AH” and “Rehab,” which suggests that the next editorial revision might consider correcting this lack of differentiation. Alternatively or additionally, separately listing of elements specific to subgroups (e.g., pediatric, military) or settings (e.g., hospital or rehab) might be considered, along with any elements specific to U.S.-based studies, such as race and ethnicity. These latter data-points may be appropriate for the U.S. approach to their population, but may not be relevant to other nations.

Overall, the degree of harmonization of study variables with the TBI-CDEs v2, between/among studies was strong, demonstrating the importance and utility of common data elements in TBI research. It confirms the potential for meta-analyses across studies, especially for CENTER-TBI and TRACK-TBI. Further refinement of CDEs should be informed by the empirical experience of these studies, retaining as a singular goal their global applicability. CENTER-TBI, TRACK-TBI, and ADAPT, along with other studies within the InTBIR Initiative, provide a platform to achieve internationalization for the next version of the TBI-CDEs.

## Supplementary Material

Supplemental data

Supplemental data

Supplemental data

Supplemental data

Supplemental data

Supplemental data

## Supplementary Material

Supplementary Table S3

Supplementary Table S3A

Supplementary Table S3B

Supplementary Table S3C
