# Supplementary material for: Common Data Elements: Critical Assessment of Harmonization between Current Multi-Center Traumatic Brain Injury Studies
Source: J Neurotrauma. 2020 May 21;37(11):1283–90. doi: 10.1089/neu.2019.6867 (PMC7249452; doi:10.1089/neu.2019.6867)
Supplement: Supplemental data [file Supp_File.pdf]

CDEs from the Acute Hospitalized domain

CDE TBI - AH							CENTER-TBI			Harmonisation	TRACK-TBI			Harmonisation	ADAPT			Harmonisation	CENTER-TBI VS TRACK-TBI		CENTER-TBI VS ADAPT		TRACK-TBI VS ADAPT	
CDE ID	CDE Name	CDE Value	Classification (e.g., Core)	Count Core	Count basic	CRF Module / Guideline	Present	CDE Value	Compatible	P+C	Present	CDE Value	Compatible	P+C	Present	CDE Value	Compatible	P+C	In Harmony	Harmonizable (cfr NIHv2)	In Harmony	Harmonizable (cfr NIHv2)	In Harmony	Harmonizable (cfr NIHv2)
C00007	Birth date		Core	1	0	Demographics	0	age: single field (Required t	0	0	0	age: single field	0	0	1	MM-DD-YYYY	1	1	1	1	1	1	1	1
C00035	Gender type	Female;Male; Unknown; Unspecified;Not reported;	Core	1	0	Demographics	1	male; female; unknown	1	1	1	male; female	1	1	1	male; female	1	1	1	1	1	1	1	1
C17396 and C18751	Sex participant or subject genotype type Sex participant or subject genotype other text	XX;XY;XXX;XYY; XXY;Other, specify; Unknown; Unspecified;	Core	1	0	Demographics	0		0	0	0		0	0	0		0	0	0	1	0	1	0	1
C00030	Race USA category	American Indian or Alaska Native; Asian;Black or African-American; Native Hawaiian or Other Pacific Islander;White; Unknown;Not Reported;	Core	1	0	Demographics	0		0	0	0		0	0	1	American Indian or Alaska Native;Asian; Black or African-American;Native Hawaiian or Other Pacific Islander; White;Unknown;Not Reported;	0	0	0	0	0	0	0	0
C00031	Race expanded category	Black African American;Black African;Black Afro Carribean; South/Central American Indian; North American Indian;Alaskan Native;Inuit;South Asian;Far Eastern Asian;Western Asian;White North American;White South American; White European; White Middle Eastern;White African;White Australian; Hawaiian;Pacific Islander;Not reported;Other; Unknown;	Core	1	0	Demographics	1	white (subcategories: north	1	1	1	white (subcategories: north american, south american, european, middle eastern, white african, australian); asian (subcategories: south asian, far eastern asian); native hawaiian/pacific islander (subcategories: hawaiian, pacific islander); alaskan native/inuit (subcategories: alaskan native, inuit); black (subcategories: african american, african, afro-caribbean)	1	1	1	American Indian or Alaska Native; Asian; Black or African American; ative Hawaiian or Other Pacific Islander; White; More than one race; Unknown or not reported	1	1	1	1	1	1	1	1
C00020	Ethnicity USA category	Hispanic or Latino; Not Hispanic or Latino;Unknown; Not reported;	Core	1	0	Demographics	0		0	0	0		0	0	1	Hispanic or Latino; Not Hispanic or Latino	0	0	0	0	0	0	0	0
C18614	Education primary caregiver year count		Core	1	0	Socioeconomic Status	1	0-30, 99=unknown	1	1	0		0	0	1		1	1	0	1	1	1	0	1
C00015	Education year count		Core	1	0	Socioeconomic Status	1	0-30; 99=unknown	1	1	1		1	1	1		1	1	1	1	1	1	1	1
C18658	Employment expanded status	Working full time; Working 20-34 hrs/week, at least minimum wage; Working less than 20 hrs/week, at least minimum wage; Temporary/odd jobs/less than minimum wage jobs;Special employment;Sick leave or maternity leave;Unemployed; Other, specify;Not in paid workforce; Unable to obtain information; Unknown;	Core	1	0	Socioeconomic Status	1	Working (35 hours or more per week); working (20 - 34 hours per week); Working (less than 20 hours per week); In working force, but currently on sick leave; Special employment / sheltered employment; Looking for work, unemployed; Unable to work; retired; Student / schoolgoing; Homemaker, keeping house; Unknown; (if in working force, specify job catergoty)	1	1	0		0	0	0		0	0	0	1	0	0	0	0
C00322	Medical history condition text		Core	1	0	Socioeconomic Status	1		1	1	1		1	1	1		1	1	1	1	1	1	1	1
C00313	Medical history condition SNOMED CT code		Core	1	0	Significant Medical History	0		0	0	0		0	0	1		1	1	0	1	0	1	0	1
C05420	TBI type	Closed;Penetrating; Blast;Crush; Unknown;	Core	1	0	Type, Place, Cause and Mechanism of Injury	1	Closed; Penetrating with open depressed skull fracture; Penetrating; Penetrating-Perforating; Penetrating–tangential; Blast; Crush.	1	1	1	closed; penetrating; blast; penetrating-perforating; penetrating-tangential; crush	1	1	1	Closed;Penetrating; Blast;Crush;	1	1	1	1	1	1	1	1
C05421	Injury ICD external cause code		Core	1	0	Type, Place, Cause and Mechanism of Injury	1	Road traffic incident; Incidental fall; Other non-intentional injury; Violence/assault; Act of mass violence; Suicide attempt; other: ____; unknown	0	0	1	Choose all that apply from code list and/or fill in appropriate ICD-9 codes	1	1	1	Choose all that apply from code list and/or fill in appropriate ICD-9 codes	1	1	1	1	1	1	1	1
C18609	ICD revision number	9;10;11;	Core	1	0	Type, Place, Cause and Mechanism of Injury	1	ICD-9	1	1	1	ICD-9	1	1	1	ICD-9	1	1	1	1	1	1	1	1
C17404	Injury elapsed time		Core	1	0	Injury Presentation - Early/Late	1	time of injury: HH-MM	1	1	1	time of injury: HH-MM	1	1	1		1	1	1	1	1	1	1	1
C01001	Glasgow Coma Scale (GCS) - motor response scale	1;2;3;4;5;6; Untestable; Unknown;	Core	1	0	Baseline Risk Assessment	1	1;2;3;4;5;6;Untestable; Unknown;	1	1	1	1;2;3;4;5;6;Untestable; Unknown;	1	1	0	1;2;3;4;5;6; Untestable;Unknown;	0	0	1	1	1	1	1	1
C01000	Glasgow Coma Scale (GCS) - eye	1;2;3;4;Untestable; Unknown;	Core	1	0	Neurological Assessment:	1	1;2;3;4;Untestable; Unknown;	1	1	1	1;2;3;4;Untestable; Unknown;	1	1	0	1;2;3;4;Untestable; Unknown;	0	0	1	1	1	1	1	1
C01053	Loss of consciousness	None;<1 minute;1-29 minutes;30-59	Core	1	0	Neurological Assessment: LOC	1	none; <1 minute; 1-29 minu	1	1	1	none; <1 minute; 1-29 minutes; 30-59 minutes;	1	1	0		0	0	1	1	0	1	0	1
C01002	Glasgow Coma Scale (GCS) - verbal response	1;2;3;4;5; Untestable; Unknown;	Core	1	0	Neurological Assessment: Glasgow Coma	1	1;2;3;4;5;Untestable; Unknown;	1	1	1	1;2;3;4;5;Untestable; Unknown;	1	1	0	1;2;3;4;5;Untestable; Unknown;	0	0	1	1	1	1	1	1
C01016	Glasgow Coma Scale (GCS) - total	3;4;5;6;7;8;9;10; 11;12;13;14;15;	Core	1	0	Neurological Assessment:	1	3;4;5;6;7;8;9;10;11;12; 13;14;15;Untestable;	1	1	1	3;4;5;6;7;8;9;10;11;12; 13;14;15;Untestable;	1	1	0	3;4;5;6;7;8;9;10;11; 12;13;14;15;	0	0	1	1	1	1	1	1
C01020	Pediatric Glasgow Coma Scale (PGCS)	1;2;3;4;Untestable; Unknown;	Core	1	0	Neurological Assessment:	0		0	0	0		0	0	1		1	1	0	0	0	0	0	0
C01021	Pediatric Glasgow Coma Scale (PGCS)	1;2;3;4;5;6; Untestable;	Core	1	0	Neurological Assessment:	0		0	0	0		0	0	1		1	1	0	0	0	0	0	0
C01022	Pediatric Glasgow Coma Scale (PGCS)	1;2;3;4;5; Untestable;	Core	1	0	Neurological Assessment:	0		0	0	0		0	0	1		1	1	0	0	0	0	0	0
C01017	Pediatric Glasgow Coma Scale (PGCS)	3;4;5;6;7;8;9;10; 11;12;13;14;15;	Core	1	0	Neurological Assessment:	0		0	0	0		0	0	1		1	1	0	0	0	0	0	0
C01055	Post traumatic amnesia duration	<1 minute;1-29 minutes;30-59	Core	1	0	Neurological Assessment: LOC	1	none; <1 minute; 1-29 minu	1	1	1	none; <1 minute; 1-29 minutes; 30-59 minutes;	1	1	0		0	0	1	1	0	1	0	1
C02500	Imaging brain assessment result	Abnormal;Normal; Not assessed;	Core	1	0	Imaging	1	Normal; Abnormal	1	1	1		1	1	1	Abnormal;Normal;Not assessed;Unknown;	1	1	1	1	1	1	1	1
C07194	Glasgow Outcome Scale Extended	1;2;3;4;5;6;7;8;	Core	1	0	Glasgow Outcome Scale Extended	1	GOSE: 1;2;3;4;5;6;7;8;	1	1	1	GOSE: 1;2;3;4;5;6;7;8;	1	1	1		1	1	1	1	1	1	1	1
C00012	Education level USA type	Never attended/Kindergar	Basic	0	1	Social Status	1	none, not currently in schoo	0	0	0		0	0	1	Grade level information for the	0	0	0	0	0	0	0	0
C00013	Education level primary caregiver USA type	Never attended/Kindergar ten only;1st Grade; 2nd Grade;3rd	Basic	0	1	Social Status	1	none, not currently in schoo	0	0	0		0	0	1	Less than 7th grade; 8th or 9th grade; 10th or 11th grade; High school graduate	0	0	0	0	0	0	0	0
C00202	Education school participation status	Going to school;On vacation from	Basic	0	1	Social Status	1	full time, diploma/degree or	0	0	0		0	0	1	Going to school;On vacation from school	1	1	0	0	0	0	0	0
C05400	Injury date time		Basic	0	1	History of Disease/Injury	1	DD-MMM-YYYY HH-MM	1	1	1	DD-MMM-YYYY HH-MM	1	1	1	MM-DD-YYYY HH-MM	1	1	1	1	1	1	1	1
C05401	Injury date reliability type	Verified;Estimated; Unknown;	Basic	0	1	History of Disease/Injury	1	Verified;Estimated; Unknown;	1	1	1		1	1	1	Verified;Estimated; Unknown (cfr MB)	1	1	1	1	1	1	1	1

CDEs from the Acute Hospitalized domain

CDE TBI - AH							CENTER-TBI			Harmonisation	TRACK-TBI			Harmonisation	ADAPT			Harmonisation
CDE ID	CDE Name	CDE Value	Classification (e.g., Core)	Count Core	Count basic	CRF Module / Guideline	Present	CDE Value	Compatible	P+C	Present	CDE Value	Compatible	P+C	Present	CDE Value	Compatible	P+C
C05419	Abusive head trauma likelihood	No concern; Possible abuse;	Basic	0	1	History of Disease/Injury	0		0	0	0		0	0	1	No concern;Possible abuse;Probable	1	1
C05422	Injury cause type	Railway accidents; Motor vehicle traffic	Basic	0	1	History of Disease/Injury	1	Road traffic incident; Incidental fall; Other non-	1	1	1	Choose all that apply from code list and/or fill in	1	1	1	Railway accidents; Motor vehicle traffic	1	1
C05423	Injury violent cause type	Robbery/assault; Interpersonal	Basic	0	1	History of Disease/Injury	1	robbery/assault; interpersonal violence	1	1	1	robbery; interpersonal violence (fight); domestic	1	1	1	Robbery/assault; Interpersonal	1	1
C05424	TBI mechanism type	Acceleration/Deceleration;Direct	Basic	0	1	History of Disease/Injury	1	High velocity trauma (acceleration/deceleration)	1	1	1	Acceleration/deceleration; Direct impact: blow to	1	1	1	Acceleration/Deceleration;Direct impact:	1	1
C05408	Hospital admission date and time		Basic	0	1	History of Disease/Injury	1	DD-MMM-YYYY; HH-MM	1	1	1	DD-MMM-YYYY; HH-MM	1	1	1	MM-DD-YYYY; HH-MM	1	1
C18397	Influence alcohol likelihood type	None;Suspected; Confirmed;	Basic	0	1	History of Disease/Injury	1	no, suspect, definite (+ blood alcohol level)	1	1	1	no, suspect, definite (+ blood alcohol level)	1	1	1	Checked on toxicology screening	1	1
C05426	Injury place occurrence type	Street/highway; Public location (e.g.	Basic	0	1	History of Disease/Injury	1	Street/highway; Public location (eg. bar, station	1	1	1	Street/highway; Home; Work/school;	1	1	1		1	1
C05427	Traffic accident self role type	Pedestrian;Cyclist; Moped/scooter;	Basic	0	1	History of Disease/Injury	1	motor vehicle occupant (driver, front seat	1	1	1	motor vehicle occupant; pedestrian; cyclist;	1	1	1	Pedestrian;Cyclist; Moped/scooter;	1	1
C05428	Traffic accident other party role	Motor vehicle; Pedestrian;Cyclist;	Basic	0	1	History of Disease/Injury	1	motor vehicle; pedestrian; cyclist; moped/scooter;	1	1	1	motor vehicle; pedestrian; cyclist; moped/scooter;	1	1	1	Motor vehicle; Pedestrian;Cyclist;	1	1
C05429	Traffic accident self alcohol influence	Suspected; Confirmed;	Basic	0	1	History of Disease/Injury	1	no, suspect, definite (+ blood alcohol level)	1	1	1	no, suspect, definite (+ blood alcohol level)	1	1	1	Suspected; Confirmed;Unknown;	1	1
C05430	Traffic accident other party alcohol	Suspected; Confirmed;	Basic	0	1	History of Disease/Injury	1	no, suspect, definite (+ blood alcohol level)	1	1	1	no, suspect, definite (+ blood alcohol level)	1	1	1	Suspected; Confirmed;Unknown;	1	1
C05431	Traffic accident self drug influence	Suspected; Confirmed;	Basic	0	1	History of Disease/Injury	1	no, suspect, definite, unknown	1	1	1	no, suspect, definite, unknown	1	1	1	Suspected; Confirmed;Unknown;	1	1
C05432	Traffic accident other party drug	Suspected; Confirmed;	Basic	0	1	History of Disease/Injury	1	no, suspect, definite, unknown	1	1	1	no, suspect, definite, unknown	1	1	1	Suspected; Confirmed;Unknown;	1	1
C05433	Protective devices use indicator	Yes;No;Unknown;	Basic	0	1	History of Disease/Injury	1	no; yes; unknown	1	1	1	yes; no; N/A; unknown	1	1	0		0	0
C05434	Vehicular protective device type	Helmet;Child safety restraint;Seat belt;	Basic	0	1	History of Disease/Injury	1	Helmet; Airbag; Seatbelt (No, Yes, Unknown)	1	1	0		0	0	0		0	0
C05435	Airbag deploy indicator	Yes;No;Not applicable;	Basic	0	1	History of Disease/Injury	1	no; yes; unknown	1	1	1		1	1	0		0	0
C00227	Military deployment injury indicator	Yes;No;Unknown;	Basic	0	1	History of Disease/Injury	0		0	0	1		1	1	0		0	0
C05437	Blast injury device and type	Improvised Explosive Device	Basic	0	1	History of Disease/Injury	0		0	0	0		0	0	0		0	0
C05438	Blast direction type	Right;Above;Below; In front;Behind;	Basic	0	1	History of Disease/Injury	0		0	0	0		0	0	0		0	0
C05439	Blast injury category	Primary blast; Secondary blast;	Basic	0	1	History of Disease/Injury	0		0	0	0		0	0	0		0	0
C05440	Blast enclose space indicator	Yes;No;Unknown;	Basic	0	1	History of Disease/Injury	0		0	0	0		0	0	0		0	0
C05441	Biological agent exposure likelihood	Suspected; Confirmed;	Basic	0	1	History of Disease/Injury	0		0	0	0		0	0	0		0	0
C05442	Chemical agent exposure likelihood	Suspected; Confirmed;	Basic	0	1	History of Disease/Injury	0		0	0	0		0	0	0		0	0
C05443	Body armor indicator	Yes;No;Unknown;	Basic	0	1	History of Disease/Injury	0		0	0	0		0	0	0		0	0
C05444	Military combat helmet type	Advanced combat helmet;Other	Basic	0	1	History of Disease/Injury	0		0	0	0		0	0	0		0	0
C00008	Age value		Basic	0	1	Classification	1	age: 3-digit number, in year	1	1	1	age: single field	1	1	1	age (cfr MB)	1	1
C01004	Pupil reactivity light left eye result	Sluggish; Nonreactive;Brisk;	Basic	0	1	Classification	1	size 1-9 mm, untestable, un	1	1	1	pupil size (1-8) + brisk, sluggish, nonreactive	1	1	1	Normal; Sluggish; Fixed; Unable to	1	1
C01003	Pupil reactivity light right eye result	Sluggish; Nonreactive;Brisk;	Basic	0	1	Classification	1	size 1-9 mm, untestable, un	1	1	1	pupil size (1-8) + brisk, sluggish, nonreactive	1	1	1	Normal; Sluggish; Fixed; Unable to	1	1
C02469	Subarachnoid hemorrhage	Present;Absent; Indeterminate;	Basic	0	1	Classification	1	no; basal; cortical	1	1	1		1	1	1	We have detailed CT imaging file that was	1	1
C02505	Marshall CT classification code	2;4;5;1;3;	Basic	0	1	Classification	1	1=diffuse injury, NVP 2=diff	1	1	1		1	1	1	Marshall score not specific for peds; we	0	0
C05453	Hypotensive episode indicator	Yes;No;Unknown; Suspected;	Basic	0	1	Second Insults	1	no; suspect; definite; unkno	1	1	1	yes; no	1	1	1	Yes;No;Unknown; Suspected;	1	1
C05457	Hypoxic episode indicator	Yes;No;Unknown; Suspected;	Basic	0	1	Second Insults	1	no; suspect; definite; unkno	1	1	1	yes; no	1	1	1	Yes;No;Unknown; Suspected;	1	1
C05459	Cardiac arrest indicator	Yes;No;Suspected; Unknown;	Basic	0	1	Second Insults	1	no; yes	1	1	1	yes; no	1	1	1	Yes;No;Unknown; Suspected;	1	1
C04803	Emergency room discharge	Medical necessity; Social	Basic	0	1	Discharge Information	1	normal CT; medical necessit	1	1	0		0	0	0		0	0
C18674	Emergency room discharge		Basic	0	1	Discharge Information	1		1	1	1		1	1	1		1	1
C04807	Vital status	Alive;Dead; Unknown;	Basic	0	1	Discharge Information	1	death; alive; unknown	1	1	1	death; alive; unknown	1	1	1	death; alive; unknown	1	1
C04804	Hospital discharge date and time		Basic	0	1	Discharge Information	1	DD-MMM-YYYY; HH-MM	1	1	1	DD-MMM-YYYY; HH-MM	1	1	1	MM-DD-YYYY	1	1
C04809	Hospital discharge destination type	Discharge to rehabilitation unit;	Basic	0	1	Discharge Information	1	discharge home; discharge	1	1	1	other hospital; rehab unit; nursing home; SNF;	1	1	1	Home; Rehabilitation facility; Skilled	1	1
C01052	Loss of consciousness	Yes;No;Suspected; Unknown;	Basic	0	1	Physical/Neurological Examination	1	no; yes -> complete GCS as	1	1	1	no; yes; suspected; unknown	1	1	1	inclusion criteria is LOC (cfr MB)	0	0
C01032	Loss of consciousness	Self-report;Witness; Clinical interview;	Basic	0	1	Physical/Neurological Examination	1	selfreport; witness; clinical i	1	1	1	patient; witness; clinical interview; medical chart;	1	1	1	inclusion criteria is LOC (cfr MB)	0	0
C01037	Post traumatic amnesia indicator	Yes;No;Suspected; Unknown;	Basic	0	1	Physical/Neurological Examination	1	no; yes; unknown	1	1	1	no; yes	1	1	0		0	0
C01033	Post traumatic amnesia verify type	Self-report;Witness; Clinical interview;	Basic	0	1	Physical/Neurological Examination	1	selfreport; witness; clinical i	1	1	1	patient; witness; clinical interview; medical chart;	1	1	0		0	0
C01041	Alteration consciousness	Yes;No;Suspected; Unknown;	Basic	0	1	Physical/Neurological Examination	1	no; yes; suspected; unknow	1	1	1	no; yes; suspected; unknov	1	1	0		0	0
C01045	Alteration of consciousness	None;<1 minute;1-29 minutes;30-59	Basic	0	1	Physical/Neurological Examination	1	none; <1 minute; 1-29 minu	1	1	1	none; <1 minute; 1-29 min	1	1	0		0	0
C01011	Glasgow Coma Scale (GCS) -	Alcohol/drugs of abuse;C-spine	Basic	0	1	Physical/Neurological Examination	0		0	0	1		1	1	1	Alcohol/drugs of abuse;C-spine injury;	1	1
C01031	Alteration of consciousness	Self-report;Witness; Clinical interview;	Basic	0	1	Physical/Neurological Examination	1	selfreport; witness; clinical i	1	1	1	patient; witness; clinical interview; medical chart;	1	1	0		0	0
C02476	Subdural hematoma	Absent; Indeterminate;	Basic	0	1	Imaging Diagnostics	1	subdural hematoma	1	1	1		1	1	1	Absent; Indeterminate;	1	1
C02480	Subdural hematoma mixed	Absent; Indeterminate;	Basic	0	1	Imaging Diagnostics	0		0	0	1		1	1	1	Absent; Indeterminate;	1	1
C02413	Contusion findings type	Non-hemorrhagic; Cortical;Subcortical;	Basic	0	1	Imaging Diagnostics	1	contusion findings	1	1	1		1	1	1	Non-hemorrhagic; Cortical;Subcortical;	1	1
C02419	Diffuse axonal injury and	Frontal - L;Parietal - R;Parietal - L;	Basic	0	1	Imaging Diagnostics	1	no; yes; unknown; number of lesions; location (corpus	1	1	1		1	1	1	Noted on CT form (cfr MB)	1	1
C05127	Intracranial surgery indicator	Yes;No;Unknown;	Basic	0	1	Surgeries and Other Procedures	1	DD-MMM-YYYY, time start H	1	1	1	Description; Date/time surgery start; Time since	1	1	1	All surgery is recorded (cfr MB)	1	1
C20338	Pediatric Glasgow Outcome Scale -	In person;By phone;From	Basic	0	1	Outcomes and End Points	0		0	0	0		0	0	1		1	1
C04802	Death date and time		Basic	0	1	End Points	1	DD-MMM-YYYY; HH-MM	1	1	1	DD-MMM-YYYY; HH-MM	1	1	1		1	1
C04808	Death cause ICD-9-CM code		Basic	0	1	End Points	1	head injury/initial injury; he	0	0	1	head injury/initial injury; head injury/secondary	0	0	1	System trauma; Increased ICP;	0	0
C18622	Brief Symptom Inventory-18		Basic	0	1	Psychiatric and Psychological	0		0	0	1	0;1;2;3;4;	0	0	0	Brief Symptom Inventory - Children	0	0

CDE ID	CENTER-TBI VS TRACK-TBI: In Harmony	CENTER-TBI VS TRACK-TBI: Harmonizable (cfr NIHv2)	CENTER-TBI VS ADAPT: In Harmony	CENTER-TBI VS ADAPT: Harmonizable (cfr NIHv2)	TRACK-TBI VS ADAPT: In Harmony	TRACK-TBI VS ADAPT: Harmonizable (cfr NIHv2)
C05419	0	0	0	0	0	0
C05422	1	1	1	1	1	1
C05423	1	1	1	1	1	1
C05424	1	1	1	1	1	1
C05408	1	1	1	1	1	1
C18397	1	1	1	1	1	1
C05426	1	1	1	1	1	1
C05427	1	1	1	1	1	1
C05428	1	1	1	1	1	1
C05429	1	1	1	1	1	1
C05430	1	1	1	1	1	1
C05431	1	1	1	1	1	1
C05432	1	1	1	1	1	1
C05433	1	1	0	1	0	1
C05434	0	1	0	1	0	1
C05435	1	1	0	1	0	1
C00227	0	1	0	0	0	0
C05437	0	0	0	0	0	0
C05438	0	0	0	0	0	0
C05439	0	0	0	0	0	0
C05440	0	0	0	0	0	0
C05441	0	0	0	0	0	0
C05442	0	0	0	0	0	0
C05443	0	0	0	0	0	0
C05444	0	0	0	0	0	0
C00008	1	1	1	1	1	1
C01004	1	1	1	1	1	1
C01003	1	1	1	1	1	1
C02469	1	1	1	1	1	1
C02505	1	1	0	1	0	1
C05453	1	1	1	1	1	1
C05457	1	1	1	1	1	1
C05459	1	1	1	1	1	1
C04803	0	1	0	1	0	1
C18674	1	1	1	1	1	1
C04807	1	1	1	1	1	1
C04804	1	1	1	1	1	1
C04809	1	1	1	1	1	1
C01052	1	1	0	0	0	0
C01032	1	1	0	0	0	0
C01037	1	1	0	0	0	0
C01033	1	1	0	0	0	0
C01041	1	1	0	0	0	0
C01045	1	1	0	0	0	0
C01011	0	1	0	1	1	1
C01031	1	1	0	0	0	0
C02476	1	1	1	1	1	1
C02480	0	1	0	1	1	1
C02413	1	1	1	1	1	1
C02419	1	1	1	1	1	1
C05127	0	1	1	1	1	1
C20338	0	0	0	0	0	0
C04802	1	1	1	1	1	1
C04808	1	1	1	1	1	1
C18622	0	0	0	0	0	0

In comparison with NIH on 20/08/2015							CENTER-TBI Present		Compatible	Harmonisation H+P+C	TRACK-TBI Present		Compatible	Harmonisation H+P+C	ADAPT Present		Compatible	Harmonisation H+P+C
TOTAL:				87			64		59	59	61		59	59	60		52	52
Core:				27			18		17	17	16		16	16	19		17	17
Basic:				60			46		42	42	45		43	43	41		35	35

	CENTER -TBI VS TRACK-TBI: In	CENTER -TBI VS TRACK-TBI: Harmonizable (cfr	CENTER -TBI VS ADAPT: In	CENTER -TBI VS ADAPT: Harmonizable (cfr	TRACK-TBI VS ADAPT: In	TRACK-TBI VS ADAPT: Harmonizable (cfr
TOTAL:	57	67	47	58	48	58
Core:	17	21	16	20	15	20
Basic:	40	46	31	38	33	38

Color Scheme of rows	
Dark orange	Excluded – non-global
Light orange	Excluded subpopulation
Green	Merged element
Yellow	Reduced element

CDEs from the Rehab domain

CDE TBI - Rehab							CENTER-TBI			Harmonisation	TRACK-TBI			Harmonisation	ADAPT			Harmonisation	CENTER-TBI VS TRACK-TBI		CENTER-TBI VS ADAPT		TRACK-TBI VS ADAPT	
CDE ID	CDE Name	CDE Value	Classification (e.g., Core)	Count Core	Count basic	CRF Module / Guideline	Present	CDE Value	Compatible	P+C	Present	CDE Value	Compatible	P+C	Present	CDE Value	Compatible	P+C	In Harmony	Harmonizable (cfr NIHv2)	In Harmony	Harmonizable (cfr NIHv2)	In Harmony	Harmonizable (cfr NIHv2)
C00007	Birth date		Core	1	0	Demographics	0	age: single field	0	0	0	age: single field	0	0	1	MM-DD-YYYY	1	1	1	1	1	1	1	1
C00035	Gender type	Female;Male; Unknown; Unspecified;Not reported;	Core	1	0	Demographics	1	male; female; unknown	1	1	1	male; female	1	1	1	male; female	1	1	1	1	1	1	1	1
C17396 and C18751	Sex participant or subject genotype type	XX;XY;XXX;XYY; XXY;Other, specify; Unknown; Unspecified;	Core	1	0	Demographics	0		0	0	0		0	0	0		0	0	0	1	0	1	0	1
C00030	Race USA category	American Indian or Alaska Native; Asian;Black or African-American; Native Hawaiian or Other Pacific Islander;White; Unknown;Not Reported;	Core	1	0	Demographics	0		0	0	0		0	0	0		0	0	0	0	0	0	0	0
C00031	Race category	Black African American;Black African;Black Afro Carribean; South/Central American Indian; North American Indian;Alaskan Native;Inuit;South Asian;Far Eastern Asian;Western Asian;White North American;White South American; White European; White Middle Eastern;White African;White Australian; Hawaiian;Pacific Islander;Not reported;Other; Unknown;	Core	1	0	Demographics	1	white (subcategories: north	1	1	1	white (subcategories: north american, south american, european, middle eastern, white african, australian); asian (subcategories: south asian, far eastern asian); native hawaiian/pacific islander (subcategories: hawaiian, pacific islander); alaskan native/inuit (subcategories: alaskan native, inuit); black (subcategories: african american, african, afro-caribbean)	1	1	1	American Indian or Alaska Native; Asian; Black or African American; ative Hawaiian or Other Pacific Islander; White; More than one race; Unknown or not reported	1	1	1	1	1	1	1	1
C00020	Ethnicity USA category	Hispanic or Latino; Not Hispanic or Latino;Unknown; Not reported;	Core	1	0	Demographics	0		0	0	0		0	0	1	Hispanic or Latino; Not Hispanic or Latino	0	0	0	0	0	0	0	0
C18614	Education primary caregiver year count		Core	1	0	Socioeconomic Status	1	0-30, 99=unknown	1	1	0		0	0	1		1	1	0	1	1	1	0	1
C00015	Education year count		Core	1	0	Socioeconomic Status	1	0-30; 99=unknown	1	1	1		1	1	1		1	1	1	1	1	1	1	1
C18658	Employment expanded status	Working full time; Working 20-34 hrs/week, at least minimum wage; Working less than 20 hrs/week, at least minimum wage; Temporary/odd jobs/less than minimum wage jobs;Special employment;Sick leave or maternity leave;Unemployed; Other, specify;Not in paid workforce; Unable to obtain information; Unknown;	Core	1	0	Socioeconomic Status	1	Working (35 hours or more per week); working (20 - 34 hours per week); Working (less than 20 hours per week); In working force, but currently on sick leave; Special employment / sheltered employment; Looking for work, unemployed; Unable to work; retired; Student / schoolgoing; Homemaker, keeping house; Unknown; (if in working force, specify job catergoty)	1	1	0		0	0	0		0	0	0	1	0	0	0	0
C00322	Medical history condition text		Core	1	0	Significant Medical History	1		1	1	1		1	1	1		1	1	1	1	1	1	1	1
C00313	Medical history condition SNOMED CT code		Core	1	0	Significant Medical History	0		0	0	0		0	0	1		1	1	0	1	0	1	0	1
C05420	TBI type	Closed;Penetrating; Blast;Crush; Unknown;	Core	1	0	Type, Place, Cause and Mechanism of Injury	1	Closed; Penetrating with open depressed skull fracture; Penetrating; Penetrating-Perforating; Penetrating–tangential; Blast; Crush.	1	1	1	closed; penetrating; blast; penetrating-perforating; penetrating-tangential; crush	1	1	1	Closed;Penetrating; Blast;Crush;	1	1	1	1	1	1	1	1
C05421	Injury ICD external cause code		Core	1	0	Type, Place, Cause and Mechanism of Injury	1	Road traffic incident; Incidental fall; Other non-intentional injury; Violence/assault; Act of mass violence; Suicide attempt; other: ____; unknown	0	0	1	Choose all that apply from code list and/or fill in appropriate ICD-9 codes	1	1	1	Choose all that apply from code list and/or fill in appropriate ICD-9 codes	1	1	1	1	1	1	1	1
C18609	ICD revision number	9;10;11;	Core	1	0	Type, Place, Cause and Mechanism of Injury	1	ICD-9	1	1	1	ICD-9	1	1	1	ICD-9	1	1	1	1	1	1	1	1
C17404	Injury elapsed time		Core	1	0	Injury Presentation - Early/Late	1	time of injury: HH-MM	1	1	1	time of injury: HH-MM	1	1	1		1	1	1	1	1	1	1	1
C01000	Glasgow Coma Scale (GCS) - eye response scale	1;2;3;4;Untestable; Unknown;	Core	1	0	Neurological Assessment: Glasgow Coma	1	1;2;3;4;Untestable; Unknown;	1	1	1	1;2;3;4;Untestable; Unknown;	1	1	0	1;2;3;4;Untestable; Unknown;	0	0	1	1	1	1	1	1
C01053	Loss of consciousness	None;<1 minute;1-29 minutes;30-59	Core	1	0	Neurological Assessment: LOC	1	none; <1 minute; 1-29 minu	1	1	1	none; <1 minute; 1-29 minutes; 30-59 minutes;	1	1	0		0	0	1	1	0	1	0	1
C01001	Glasgow Coma Scale (GCS) - motor	1;2;3;4;5;6; Untestable;	Core	1	0	Neurological Assessment:	1	1;2;3;4;5;6;Untestable; Unknown;	1	1	1	1;2;3;4;5;6;Untestable; Unknown;	1	1	0	1;2;3;4;5;6; Untestable;Unknown;	0	0	1	1	1	1	1	1
C01002	Glasgow Coma Scale (GCS) - verbal response	1;2;3;4;5; Untestable; Unknown;	Core	1	0	Neurological Assessment: Glasgow Coma	1	1;2;3;4;5;Untestable; Unknown;	1	1	1	1;2;3;4;5;Untestable; Unknown;	1	1	0	1;2;3;4;5;Untestable; Unknown;	0	0	1	1	1	1	1	1
C01016	Glasgow Coma Scale (GCS) - total	3;4;5;6;7;8;9;10; 11;12;13;14;15;	Core	1	0	Neurological Assessment:	1	3;4;5;6;7;8;9;10;11;12; 13;14;15;Untestable;	1	1	1	3;4;5;6;7;8;9;10;11;12; 13;14;15;Untestable;	1	1	0	3;4;5;6;7;8;9;10;11; 12;13;14;15;	0	0	1	1	1	1	1	1
C01020	Pediatric Glasgow Coma Scale (PGCS)	1;2;3;4;Untestable; Unknown;	Core	1	0	Neurological Assessment:	0		0	0	0		0	0	1		1	1	0	0	0	0	0	0
C01021	Pediatric Glasgow Coma Scale (PGCS)	1;2;3;4;5;6; Untestable;	Core	1	0	Neurological Assessment:	0		0	0	0		0	0	1		1	1	0	0	0	0	0	0
C01022	Pediatric Glasgow Coma Scale (PGCS)	1;2;3;4;5; Untestable;	Core	1	0	Neurological Assessment:	0		0	0	0		0	0	1		1	1	0	0	0	0	0	0
C01017	Pediatric Glasgow Coma Scale (PGCS)	3;4;5;6;7;8;9;10; 11;12;13;14;15;	Core	1	0	Neurological Assessment:	0		0	0	0		0	0	1		1	1	0	0	0	0	0	0
C01055	Post traumatic amnesia duration	<1 minute;1-29 minutes;30-59	Core	1	0	Neurological Assessment: LOC	1	none; <1 minute; 1-29 minu	1	1	1	none; <1 minute; 1-29 minutes; 30-59 minutes;	1	1	0		0	0	1	1	0	1	0	1
C02500	Imaging brain assessment result	Abnormal;Normal; Not assessed;	Core	1	0	Imaging	1	Normal; Abnormal	1	1	1		1	1	1		1	1	1	1	1	1	1	1
C07194	Glasgow Outcome Scale Extended	1;2;3;4;5;6;7;8;	Core	1	0	Glasgow Outcome Scale Extended	1	GOSE: 1;2;3;4;5;6;7;8;	1	1	1	GOSE: 1;2;3;4;5;6;7;8;	1	1	1		1	1	1	1	1	1	1	1
C00012	Education level USA type	Never attended/Kindergar	Basic	0	1	Social Status	1	none, not currently in schoo	0	0	0		0	0	0		0	0	0	0	0	0	0	0
C00013	Education level primary caregiver USA type	Never attended/Kindergar ten only;1st Grade; 2nd Grade;3rd	Basic	0	1	Social Status	1	none, not currently in schoo	0	0	0		0	0	1	Less than 7th grade; 8th or 9th grade; 10th or 11th grade; High school graduate	0	0	0	0	0	0	0	0
C00202	Education school participation status	Going to school;On vacation from	Basic	0	1	Social Status	1	full time, diploma/degree or	0	0	0		0	0	1		1	1	0	0	0	0	0	0
C00207	Marital or partner status	Never married; Married;Domestic	Basic	0	1	Socioeconomic Status	1	never been married; marrie	1	1	1	Never married; Married; Domestic partnership;	1	1	0		0	0	1	1	0	0	0	0
C05400	Injury date time		Basic	0	1	History of Disease/Injury	1	DD-MMM-YYYY HH-MM	1	1	1	DD-MMM-YYYY HH-MM	1	1	1	MM-DD-YYYY HH-MM	1	1	1	1	1	1	1	1
C05422	Injury cause type	Railway accidents; Motor vehicle traffic	Basic	0	1	History of Disease/Injury	1	Road traffic incident; Incidental fall; Other non-	1	1	1	Choose all that apply from code list and/or fill in	1	1	1	Railway accidents; Motor vehicle traffic	1	1	1	1	1	1	1	1
C05423	Injury violent cause type	Robbery/assault; Interpersonal	Basic	0	1	History of Disease/Injury	1	robbery/assault; interpersonal violence	1	1	1	robbery; interpersonal violence (fight); domestic	1	1	1		1	1	1	1	1	1	1	1
C05424	TBI mechanism type	Acceleration/Deceleration;Direct	Basic	0	1	History of Disease/Injury	1	High velocity trauma (acceleration/deceleration)	1	1	1	Acceleration/deceleration; Direct impact: blow to	1	1	1	Acceleration/Deceleration;Direct impact:	1	1	1	1	1	1	1	1
C18397	Influence alcohol likelihood type	None;Suspected; Confirmed;	Basic	0	1	History of Disease/Injury	1	no, suspect, definite (+ blood alcohol level)	1	1	1	no, suspect, definite (+ blood alcohol level)	1	1	1		1	1	1	1	1	1	1	1

CDEs from the Rehab domain

CDE TBI - Rehab							CENTER-TBI			Harmonisation	TRACK-TBI			Harmonisation	ADAPT			Harmonisation	CENTER-TBI VS TRACK-TBI		CENTER-TBI VS ADAPT		TRACK-TBI VS ADAPT	
CDE ID	CDE Name	CDE Value	Classification (e.g., Core)	Count Core	Count basic	CRF Module / Guideline	Present	CDE Value	Compatible	P+C	Present	CDE Value	Compatible	P+C	Present	CDE Value	Compatible	P+C	In Harmony	Harmonizable (cfr NIHv2)	In Harmony	Harmonizable (cfr NIHv2)	In Harmony	Harmonizable (cfr NIHv2)
C05426	Injury place	Street/highway;	Basic	0	1	History of	1	Street/highway; Public	1	1	1	Street/highway; Home;	1	1	1		1	1	1	1	1	1	1	1
C05427	Traffic accident self	Pedestrian;Cyclist;	Basic	0	1	History of	1	motor vehicle occupant	1	1	1	motor vehicle occupant;	1	1	1		1	1	1	1	1	1	1	1
C05428	Traffic accident	Motor vehicle;	Basic	0	1	History of	1	motor vehicle; pedestrian;	1	1	1	motor vehicle; pedestrian;	1	1	1		1	1	1	1	1	1	1	1
C05429	Traffic accident self	Suspected;	Basic	0	1	History of	1	no, suspect, definite (+	1	1	1	no, suspect, definite (+	1	1	1		1	1	1	1	1	1	1	1
C05430	Traffic accident	Suspected;	Basic	0	1	History of	1	no, suspect, definite (+	1	1	1	no, suspect, definite (+	1	1	1		1	1	1	1	1	1	1	1
C05431	Traffic accident self	Suspected;	Basic	0	1	History of	1	no, suspect, definite,	1	1	1	no, suspect, definite,	1	1	1		1	1	1	1	1	1	1	1
C05432	Traffic accident	Suspected;	Basic	0	1	History of	1	no, suspect, definite,	1	1	1	no, suspect, definite,	1	1	1		1	1	1	1	1	1	1	1
C05433	Protective devices	Yes;No;Unknown;	Basic	0	1	History of	1	no; yes; N/A	1	1	1	yes; no; N/A; unknown	1	1	0		0	0	1	1	0	1	0	1
C05434	Vehicular protective	Helmet;Child safety	Basic	0	1	History of	1	Helmet; Airbag; Seatbelt	1	1	0		0	0	0		0	0	0	1	0	1	0	1
C05435	Airbag deploy	Yes;No;Not	Basic	0	1	History of	1	no; yes; N/A	1	1	1		1	1	0		0	0	1	1	0	1	0	1
C00227	Military deployment	Yes;No;Unknown;	Basic	0	1	History of	0		0	0	1		1	1	0		0	0	0	1	0	0	0	0
C05437	Blast injury device	Improvised	Basic	0	1	History of	0		0	0	0		0	0	0		0	0	0	0	0	0	0	0
C05438	Blast direction type	Right;Above;Below;	Basic	0	1	History of	0		0	0	0		0	0	0		0	0	0	0	0	0	0	0
C05439	Blast injury	Primary blast;	Basic	0	1	History of	0		0	0	0		0	0	0		0	0	0	0	0	0	0	0
C05440	Blast enclose space	Yes;No;Unknown;	Basic	0	1	History of	0		0	0	0		0	0	0		0	0	0	0	0	0	0	0
C05441	Biological agent	Suspected;	Basic	0	1	History of	0		0	0	0		0	0	0		0	0	0	0	0	0	0	0
C05442	Chemical agent	Suspected;	Basic	0	1	History of	0		0	0	0		0	0	0		0	0	0	0	0	0	0	0
C05443	Body armor	Yes;No;Unknown;	Basic	0	1	History of	0		0	0	0		0	0	0		0	0	0	0	0	0	0	0
C05444	Military combat	Advanced combat	Basic	0	1	History of	0		0	0	0		0	0	0		0	0	0	0	0	0	0	0
C02469	Subarachnoid	Present;Absent;	Basic	0	1	Classification	1	no; basal; cortical	1	1	1		1	1	1		1	1	1	1	1	1	1	1
C04804	Hospital discharge		Basic	0	1	Discharge	1	DD-MMM-YYYY; HH-MM	1	1	1	DD-MMM-YYYY; HH-MM	1	1	1	MM-DD-YYYY	1	1	1	1	1	1	1	1
C04809	Hospital discharge	Discharge to	Basic	0	1	Discharge	1	discharge home; discharge (	1	1	1	other hospital; rehab unit;	1	1	1	Home; Rehabilitation	1	1	1	1	1	1	1	1
C01052	Loss of consciousness	Yes;No;Suspected; Unknown;	Basic	0	1	Physical/Neurological Examination	1	no; yes -> complete GCS as	1	1	1	no; yes; suspected; unknown	1	1	0		0	0	1	1	0	0	0	0
C01032	Loss of consciousness verification type	Self-report;Witness; Clinical interview; Medical chart;Not	Basic	0	1	Physical/Neurological Examination	1	selfreport; witness; clinical i	1	1	1	patient; witness; clinical interview; medical chart; not available	1	1	0		0	0	1	1	0	0	0	0
C01037	Post traumatic	Yes;No;Suspected;	Basic	0	1	Physical/Neurologi	1	no; yes; unknown	1	1	1	no; yes	1	1	0		0	0	1	1	0	0	0	0
C01033	Post traumatic	Self-report;Witness;	Basic	0	1	Physical/Neurologi	1	selfreport; witness; clinical i	1	1	1	patient; witness; clinical	1	1	0		0	0	1	1	0	0	0	0
C01041	Alteration	Yes;No;Suspected;	Basic	0	1	Physical/Neurologi	1	no; yes; suspected; unknow	1	1	1	no; yes; suspected; unknov	1	1	0		0	0	1	1	0	0	0	0
C01045	Alteration of	None;<1 minute;1-	Basic	0	1	Physical/Neurologi	1	none; <1 minute; 1-29 minu	1	1	1	none; <1 minute; 1-29 min	1	1	0		0	0	1	1	0	0	0	0
C01031	Alteration of	Self-report;Witness;	Basic	0	1	Physical/Neurologi	1	selfreport; witness; clinical i	1	1	1	patient; witness; clinical	1	1	0		0	0	1	1	0	0	0	0
C01004	Pupil reactivity light	Sluggish;	Basic	0	1	Neurological	1	size 1-9 mm, untestable, un	1	1	1	pupil size (1-8) + brisk,	1	1	1	Normal; Sluggish;	1	1	1	1	1	1	1	1
C01003	Pupil reactivity light	Sluggish;	Basic	0	1	Neurological	1	size 1-9 mm, untestable, un	1	1	1	pupil size (1-8) + brisk,	1	1	1	Normal; Sluggish;	1	1	1	1	1	1	1	1
C01006	Pupil left eye	mm	Basic	0	1	Neurological	1	size 1-9 mm, untestable, un	1	1	1	pupil size (1-8) + brisk,	1	1	1	Size in mm	1	1	1	1	1	1	1	1
C01005	Pupil right eye	mm	Basic	0	1	Neurological	1	size 1-9 mm, untestable, un	1	1	1	pupil size (1-8) + brisk,	1	1	1	Size in mm	1	1	1	1	1	1	1	1
C01024	Pupil shape left eye	Round;Oval;	Basic	0	1	Neurological	0		0	0	1	pupil size (1-8) + brisk,	1	1	1	Round; Oval; Unable	1	1	0	1	0	1	1	1
C01023	Pupil shape right	Round;Oval;	Basic	0	1	Neurological	0		0	0	1	pupil size (1-8) + brisk,	1	1	1	Round; Oval; Unable	1	1	0	1	0	1	1	1
C02476	Subdural	Absent;	Basic	0	1	Imaging	1	subdural hematoma	1	1	1		1	1	1		1	1	1	1	1	1	1	1
C02480	Subdural	Absent;	Basic	0	1	Imaging	0		0	0	1		1	1	1		1	1	0	1	0	1	1	1
C02413	Contusion findings	Non-hemorrhagic;	Basic	0	1	Imaging	1	contusion findings	1	1	1		1	1	1		1	1	1	1	1	1	1	1
C02419	Diffuse axonal	Frontal - L;Parietal	Basic	0	1	Imaging	1	no; yes; unknown; number	1	1	1		1	1	1		1	1	1	1	1	1	1	1
C05127	Intracranial surgery	Yes;No;Unknown;	Basic	0	1	Surgeries and	1	DD-MMM-YYYY, time start H	1	1	1	Description; Date/time	1	1	1		1	1	0	1	1	1	1	1
C05130	Therapy or	Speech therapy;	Basic	0	1	Post	1	Physical therapy;	1	1	1		1	1	0		0	0	1	1	0	1	0	1
C05131	Therapy		Basic	0	1	Post	0		0	0	0		0	0	0		0	0	0	1	0	1	0	1
C05132	Therapy or		Basic	0	1	Post	1	1=none; 2=only follow-up,	1	1	1		1	1	0		0	0	1	1	0	1	0	1
C05133	Therapy or		Basic	0	1	Post	1	2-4 weeks; 3 months; 6	1	1	1		1	1	0		0	0	1	1	0	1	0	1
C05134	Therapy		Basic	0	1	Post	1	DD-MMM-YYYY	1	1	1		1	1	0		0	0	1	1	0	1	0	1
C05135	Therapy		Basic	0	1	Post	1	DD-MMM-YYYY	1	1	1		1	1	0		0	0	1	1	0	1	0	1
C05136	Therapy	Yes;No;Unknown;	Basic	0	1	Post	1	Yes;No;Unknown;	1	1	1		1	1	0		0	0	1	1	0	1	0	1
C20338	Pediatric Glasgow	In person;By	Basic	0	1	Post	0		0	0	0		0	0	1		1	1	0	0	0	0	0	0
C04802	Death date and		Basic	0	1	End Points	1	DD-MMM-YYYY; HH-MM	1	1	1	DD-MMM-YYYY; HH-MM	1	1	1	MM-DD-YYYY; HH-	1	1	1	1	1	1	1	1
C04808	Death cause ICD-9-		Basic	0	1	End Points	1	head injury/initial injury; he	0	0	1	head injury/initial injury;	0	0	1		0	0	1	1	1	1	1	1
C04814	Residence type and	Hospital;Nursing	Basic	0	1	Outcomes and	1	no; out-patient	1	1	1		1	1	0		0	0	1	1	0	1	0	1
C07442	Satisfaction with	1;2;3;4;5;6;7;	Basic	0	1	Outcomes and	0		0	0	1		1	1	0		0	0	0	1	0	0	0	0
C18622	Brief Symptom		Basic	0	1	Outcomes and	0		0	0	1		0	0	0		0	0	0	0	0	0	0	0
C19546 + C19547 + C07112	Craig Handicap and Assessment Reporting Technique (CHART-	Less than 10,000; 10,000–15,000; 15,000–20,000; 20,000–25,000;	Basic	0	1	Outcomes and End Points	0		0	0	1		1	1	0		0	0	0	1	0	0	0	0

In comparison with NIH on 20/08/2015							CENTER-TBI Present		Compatibl	Harmonisation H+P+C	TRACK-TBI Present		Compatibl	Harmonisation H+P+C	ADAPT Present		Compatibl	Harmonisation H+P+C	CENTER -TBI VS TRACK-TBI In	Harmonizable (cfr	CENTER -TBI VS ADAPT In	Harmonizable (cfr	TRACK-TBI VS ADAPT In	Harmonizable (cfr
TOTAL:				90			64		59	59	65		63	63	49		46	46	58	71	41	59	43	59
Core:				27			18		17	17	16		16	16	18		17	17	17	21	16	20	15	20
Basic:				63			46		42	42	49		47	47	31		29	29	41	50	25	39	28	39

Color Scheme of rows	
Dark orange	Excluded – non-global
Light orange	Excluded subpopulation
Green	Merged element
Yellow	Reduced element

Basic CDEs common to Acute Hospitalized and Rehab domains

CDE - Common Basic Elements							CENTER-TBI			Harmonisation	TRACK-TBI			Harmonisation	ADAPT			Harmonisation
CDE ID	CDE Name	CDE Value	Classification (e.g., Core)	Count Core	Count basic	CRF Module / Guideline	Present	CDE Value	Compatible	P+C	Present	CDE Value	Compatible	P+C	Present	CDE Value	Compatible	P+C
C00012	Education level USA	Never	Basic	0	1	Social Status	1	none, not currently in schoo	0	0	0		0	0	1	Grade level	0	0
C00013	Education level	Never	Basic	0	1	Social Status	1	none, not currently in schoo	0	0	0		0	0	1	Less than 7th grade;	0	0
C00202	Education school	Going to school;On	Basic	0	1	Social Status	1	full time, diploma/degree or	1	0	0		0	0	1	Going to school;On	1	1
C05400	Injury date time		Basic	0	1	History of	1	DD-MMM-YYYY HH-MM	1	1	1	DD-MMM-YYYY HH-MM	1	1	1	MM-DD-YYYY HH-MM	1	1
C05422	Injury cause type	Railway accidents;	Basic	0	1	History of	1	Road traffic incident;	1	1	1	Choose all that apply from	1	1	1	Railway accidents;	1	1
C05423	Injury violent cause	Robbery/assault;	Basic	0	1	History of	1	robbery/assault;	1	1	1	robbery; interpersonal	1	1	1	Robbery/assault;	1	1
C05424	TBI mechanism	Acceleration/Decele	Basic	0	1	History of	1	High velocity trauma	1	1	1	Acceleration/deceleration;	1	1	1	Acceleration/Decelera	1	1
C18397	Influence alcohol	None;Suspected;	Basic	0	1	History of	1	no, suspect, definite (+	1	1	1	no, suspect, definite (+	1	1	1	Checked on	1	1
C05426	Injury place	Street/highway;	Basic	0	1	History of	1	Street/highway; Public	1	1	1	Street/highway; Home;	1	1	1		1	1
C05427	Traffic accident self	Pedestrian;Cyclist;	Basic	0	1	History of	1	motor vehicle occupant	1	1	1	motor vehicle occupant;	1	1	1	Pedestrian;Cyclist;	1	1
C05428	Traffic accident	Motor vehicle;	Basic	0	1	History of	1	motor vehicle; pedestrian;	1	1	1	motor vehicle; pedestrian;	1	1	1	Motor vehicle;	1	1
C05429	Traffic accident self	Suspected;	Basic	0	1	History of	1	no, suspect, definite (+	1	1	1	no, suspect, definite (+	1	1	1	Suspected;	1	1
C05430	Traffic accident	Suspected;	Basic	0	1	History of	1	no, suspect, definite (+	1	1	1	no, suspect, definite (+	1	1	1	Suspected;	1	1
C05431	Traffic accident self	Suspected;	Basic	0	1	History of	1	no, suspect, definite,	1	1	1	no, suspect, definite,	1	1	1	Suspected;	1	1
C05432	Traffic accident	Suspected;	Basic	0	1	History of	1	no, suspect, definite,	1	1	1	no, suspect, definite,	1	1	1	Suspected;	1	1
C05433	Protective devices	Yes;No;Unknown;	Basic	0	1	History of	1	no; yes; unknown	1	1	1	yes; no; N/A; unknown	1	1	0		0	0
C05434	Vehicular protective	Helmet;Child safety	Basic	0	1	History of	1	Helmet; Airbag; Seatbelt	1	1	0		0	0	0		0	0
C05435	Airbag deploy	Yes;No;Not	Basic	0	1	History of	1	no; yes; unknown	1	1	1		1	1	0		0	0
C00227	Military deployment	Yes;No;Unknown;	Basic	0	1	History of	0		0	0	1		1	1	0		0	0
C05437	Blast injury device	Improvised	Basic	0	1	History of	0		0	0	0		0	0	0		0	0
C05438	Blast direction type	Right;Above;Below;	Basic	0	1	History of	0		0	0	0		0	0	0		0	0
C05439	Blast injury	Primary blast;	Basic	0	1	History of	0		0	0	0		0	0	0		0	0
C05440	Blast enclose space	Yes;No;Unknown;	Basic	0	1	History of	0		0	0	0		0	0	0		0	0
C05441	Biological agent	Suspected;	Basic	0	1	History of	0		0	0	0		0	0	0		0	0
C05442	Chemical agent	Suspected;	Basic	0	1	History of	0		0	0	0		0	0	0		0	0
C05443	Body armor	Yes;No;Unknown;	Basic	0	1	History of	0		0	0	0		0	0	0		0	0
C05444	Military combat	Advanced combat	Basic	0	1	History of	0		0	0	0		0	0	0		0	0
C01004	Pupil reactivity light	Sluggish;	Basic	0	1	Classification	1	size 1-9 mm, untestable, un	1	1	1	pupil size (1-8) + brisk,	1	1	1	Normal; Sluggish;	1	1
C01003	Pupil reactivity light	Sluggish;	Basic	0	1	Classification	1	size 1-9 mm, untestable, un	1	1	1	pupil size (1-8) + brisk,	1	1	1	Normal; Sluggish;	1	1
C02469	Subarachnoid	Present;Absent;	Basic	0	1	Classification	1	no; basal; cortical	1	1	1		1	1	1	We have detailed CT	1	1
C04804	Hospital discharge		Basic	0	1	Discharge	1	DD-MMM-YYYY; HH-MM	1	1	1	DD-MMM-YYYY; HH-MM	1	1	1	MM-DD-YYYY	1	1
C04809	Hospital discharge	Discharge to	Basic	0	1	Discharge	1	discharge home; discharge	1	1	1	other hospital; rehab unit;	1	1	1	Home; Rehabilitation	1	1
C01052	Loss of	Yes;No;Suspected;	Basic	0	1	Physical/Neurologi	1	no; yes -> complete GCS as	1	1	1	no; yes; suspected;	1	1	1	inclusion criteria is	0	0
C01032	Loss of	Self-report;Witness;	Basic	0	1	Physical/Neurologi	1	selfreport; witness; clinical i	1	1	1	patient; witness; clinical	1	1	1	inclusion criteria is	0	0
C01037	Post traumatic	Yes;No;Suspected;	Basic	0	1	Physical/Neurologi	1	no; yes; unknown	1	1	1	no; yes	1	1	0		0	0
C01033	Post traumatic	Self-report;Witness;	Basic	0	1	Physical/Neurologi	1	selfreport; witness; clinical i	1	1	1	patient; witness; clinical	1	1	0		0	0
C01041	Alteration	Yes;No;Suspected;	Basic	0	1	Physical/Neurologi	1	no; yes; suspected; unknow	1	1	1	no; yes; suspected; unknov	1	1	0		0	0
C01045	Alteration of	None;<1 minute;1-	Basic	0	1	Physical/Neurologi	1	none; <1 minute; 1-29 minu	1	1	1	none; <1 minute; 1-29 min	1	1	0		0	0
C01031	Alteration of	Self-report;Witness;	Basic	0	1	Physical/Neurologi	1	selfreport; witness; clinical i	1	1	1	patient; witness; clinical	1	1	0		0	0
C02476	Subdural	Absent;	Basic	0	1	Imaging	1	subdural hematoma	1	1	1		1	1	1	Absent;	1	1
C02480	Subdural	Absent;	Basic	0	1	Imaging	0		0	0	1		1	1	1	Absent;	1	1
C02413	Contusion findings	Non-hemorrhagic;	Basic	0	1	Imaging	1	contusion findings	1	1	1		1	1	1	Non-hemorrhagic;	1	1
C02419	Diffuse axonal	Frontal - L;Parietal	Basic	0	1	Imaging	1	no; yes; unknown; number	1	1	1		1	1	1	Noted on CT form	1	1
C05127	Intracranial surgery	Yes;No;Unknown;	Basic	0	1	Surgeries and	1	DD-MMM-YYYY, time start H	1	1	1	Description; Date/time	1	1	1	All surgery is	1	1
C20338	Pediatric Glasgow	In person;By	Basic	0	1	Outcomes and	0		0	0	0		0	0	1		1	1
C04802	Death date and		Basic	0	1	End Points	1	DD-MMM-YYYY; HH-MM	1	1	1	DD-MMM-YYYY; HH-MM	1	1	1		1	1
C04808	Death cause ICD-9-		Basic	0	1	End Points	1	head injury/initial injury; he	0	0	1	head injury/initial injury;	0	0	1	System trauma;	0	0
C18622	Brief Symptom		Basic	0	1	Psychiatric and	0		0	0	1	0;1;2;3;4;	0	0	0	Brief Symptom	0	0

	CENTER-TBI VS TRACK-TBI		CENTER-TBI VS ADAPT		TRACK-TBI VS ADAPT	
CDE ID	In Harmony	Harmonizable (cfr NIHv2)	In Harmony	Harmonizable (cfr NIHv2)	In Harmony	Harmonizable (cfr NIHv2)
C00012	0	0	0	0	0	0
C00013	0	0	0	0	0	0
C00202	0	0	0	0	0	0
C05400	1	1	1	1	1	1
C05422	1	1	1	1	1	1
C05423	1	1	1	1	1	1
C05424	1	1	1	1	1	1
C18397	1	1	1	1	1	1
C05426	1	1	1	1	1	1
C05427	1	1	1	1	1	1
C05428	1	1	1	1	1	1
C05429	1	1	1	1	1	1
C05430	1	1	1	1	1	1
C05431	1	1	1	1	1	1
C05432	1	1	1	1	1	1
C05433	1	1	0	1	0	1
C05434	0	1	0	1	0	1
C05435	1	1	0	1	0	1
C00227	0	1	0	0	0	0
C05437	0	0	0	0	0	0
C05438	0	0	0	0	0	0
C05439	0	0	0	0	0	0
C05440	0	0	0	0	0	0
C05441	0	0	0	0	0	0
C05442	0	0	0	0	0	0
C05443	0	0	0	0	0	0
C05444	0	0	0	0	0	0
C01004	1	1	1	1	1	1
C01003	1	1	1	1	1	1
C02469	1	1	1	1	1	1
C04804	1	1	1	1	1	1
C04809	1	1	1	1	1	1
C01052	1	1	0	0	0	0
C01032	1	1	0	0	0	0
C01037	1	1	0	0	0	0
C01033	1	1	0	0	0	0
C01041	1	1	0	0	0	0
C01045	1	1	0	0	0	0
C01031	1	1	0	0	0	0
C02476	1	1	1	1	1	1
C02480	0	1	0	1	1	1
C02413	1	1	1	1	1	1
C02419	1	1	1	1	1	1
C05127	0	1	1	1	1	1
C20338	0	0	0	0	0	0
C04802	1	1	1	1	1	1
C04808	1	1	1	1	1	1
C18622	0	0	0	0	0	0

In comparison with NIH on 20/08/2015										Harmonisation				Harmonisation				Harmonisation
							Present		Compatible	H+P+C	Present		Compatible	H+P+C	Present		Compatible	H+P+C
Basic:					48		36		33	32	35		33	33	30		25	25

CENTER -TBI VS TRACK-TBI		CENTER -TBI VS ADAPT		TRACK-TBI VS ADAPT	
In	Harmonizable (cfr	In	Harmonizable (cfr	In	Harmonizable (cfr
31	35	23	27	24	27

Color Scheme of rows	
Dark orange	Excluded – non-global
Light orange	Excluded subpopulation
Green	Merged element
Yellow	Reduced element

Basic CDEs unique to Acute Hospitalized domain

CDE - Unique AH							CENTER-TBI			Harmonisation
CDE ID	CDE Name	CDE Value	Classification (e.g., Core)	Count Core	Count basic	CRF Module / Guideline	Present	CDE Value	Compatible	P+C
C05401	Injury date reliability type	Verified;Estimated; Unknown;	Basic	0	1	History of Disease/Injury	1	Verified;Estimated; Unknown;	1	1
C05419	Abusive head trauma likelihood	No concern; Possible abuse;	Basic	0	1	History of Disease/Injury	0		0	0
C05408	Hospital admission date and time		Basic	0	1	History of Disease/Injury	1	DD-MMM-YYYY; HH-MM	1	1
C00008	Age value		Basic	0	1	Classification	1	age: 3-digit number, in year	1	1
C02505	Marshall CT classification code	2;4;5;1;3;	Basic	0	1	Classification	1	1=diffuse injury, NVP 2=diff	1	1
C05453	Hypotensive episode indicator	Yes;No;Unknown; Suspected;	Basic	0	1	Second Insults	1	no; suspect; definite; unkno	1	1
C05457	Hypoxic episode indicator	Yes;No;Unknown; Suspected;	Basic	0	1	Second Insults	1	no; suspect; definite; unkno	1	1
C05459	Cardiac arrest indicator	Yes;No;Suspected; Unknown;	Basic	0	1	Second Insults	1	no; yes	1	1
C04803	Emergency room discharge	Medical necessity; Social	Basic	0	1	Discharge Information	1	normal CT; medical necessit	1	1
C18674	Emergency room discharge		Basic	0	1	Discharge Information	1		1	1
C04807	Vital status	Alive;Dead; Unknown;	Basic	0	1	Discharge Information	1	death; alive; unknown	1	1
C01011	Glasgow Coma Scale (GCS) -	Alcohol/drugs of abuse;C-spine	Basic	0	1	Physical/Neurological Examination	0		0	0

CDE ID	TRACK-TBI Present	TRACK-TBI CDE Value	TRACK-TBI Compatible	Harmonisation P+C	ADAPT Present	ADAPT CDE Value	ADAPT Compatible	Harmonisation P+C
C05401	1		1	1	1	Verified;Estimated; Unknown (cfr MB)	1	1
C05419	0		0	0	1	No concern;Possible abuse;Probable	1	1
C05408	1	DD-MMM-YYYY; HH-MM	1	1	1	MM-DD-YYYY; HH-MM	1	1
C00008	1	age: single field	1	1	1	age (cfr MB)	1	1
C02505	1		1	1	1	Marshall score not specific for peds; we	0	0
C05453	1	yes; no	1	1	1	Yes;No;Unknown; Suspected;	1	1
C05457	1	yes; no	1	1	1	Yes;No;Unknown; Suspected;	1	1
C05459	1	yes; no	1	1	1	Yes;No;Unknown; Suspected;	1	1
C04803	0		0	0	0		0	0
C18674	1		1	1	1		1	1
C04807	1	death; alive; unknown	1	1	1	death; alive; unknown	1	1
C01011	1		1	1	1	Alcohol/drugs of abuse;C-spine injury;	1	1

CDE ID	CENTER-TBI VS TRACK-TBI In Harmony	CENTER-TBI VS TRACK-TBI Harmonizable (cfr NIHv2)	CENTER-TBI VS ADAPT In Harmony	CENTER-TBI VS ADAPT Harmonizable (cfr NIHv2)	TRACK-TBI VS ADAPT In Harmony	TRACK-TBI VS ADAPT Harmonizable (cfr NIHv2)
C05401	1	1	1	1	1	1
C05419	0	0	0	0	0	0
C05408	1	1	1	1	1	1
C00008	1	1	1	1	1	1
C02505	1	1	0	1	0	1
C05453	1	1	1	1	1	1
C05457	1	1	1	1	1	1
C05459	1	1	1	1	1	1
C04803	0	1	0	1	0	1
C18674	1	1	1	1	1	1
C04807	1	1	1	1	1	1
C01011	1	1	0	1	1	1

In comparison with NIH on 20/08/2015							CENTER-TBI Presen		Compatibl	Harmonisation H+P+C	TRACK-TBI Presen		Compatibl	Harmonisation H+P+C	ADAPT Presen		Compatibl	Harmonisation H+P+C
TOTAL:				12			10		10	10	10		10	10	11		10	10
Basic:				12			10		10	10	10		10	10	11		10	10

	CENTER -TBI VS TRACK-TBI In	CENTER -TBI VS TRACK-TBI Harmonizable (cfr	CENTER -TBI VS ADAPT In	CENTER -TBI VS ADAPT Harmonizable (cfr	TRACK-TBI VS ADAPT In	TRACK-TBI VS ADAPT Harmonizable (cfr
TOTAL:	10	11	8	11	9	11
Basic:	10	11	8	11	9	11

Color Scheme of rows	
Dark orange	Excluded – non-global
Light orange	Excluded subpopulation
Green	Merged element
Yellow	Reduced element

Basic CDEs unique to Rehab domain

CDE - Unique Rehab							CENTER-TBI			Harmonisation	TRACK-TBI			Harmonisation	ADAPT			Harmonisation
CDE ID	CDE Name	CDE Value	Classification (e.g., Core)	Count Core	Count basic	CRF Module / Guideline	Present	CDE Value	Compatible	P+C	Present	CDE Value	Compatible	P+C	Present	CDE Value	Compatible	P+C
C00207	Marital or partner status	Never married; Married;Domestic	Basic	0	1	Socioeconomic Status	1	never been married; marrie	1	1	1	Never married; Married; Domestic partnership;	1	1	0		0	0
C01006	Pupil left eye measurement	mm	Basic	0	1	Neurological Assessment:	1	size 1-9 mm, untestable, un	1	1	1	pupil size (1-8) + brisk, sluggish, nonreactive	1	1	1	Size in mm	1	1
C01005	Pupil right eye measurement	mm	Basic	0	1	Neurological Assessment:	1	size 1-9 mm, untestable, un	1	1	1	pupil size (1-8) + brisk, sluggish, nonreactive	1	1	1	Size in mm	1	1
C01024	Pupil shape left eye type	Round;Oval; Unknown;	Basic	0	1	Neurological Assessment:	0		0	0	1	pupil size (1-8) + brisk, sluggish, nonreactive	1	1	1	Round; Oval; Unable to asses	1	1
C01023	Pupil shape right eye type	Round;Oval; Unknown;	Basic	0	1	Neurological Assessment:	0		0	0	1	pupil size (1-8) + brisk, sluggish, nonreactive	1	1	1	Round; Oval; Unable to asses	1	1
C05130	Therapy or rehabilitation type	Speech therapy; Occupational	Basic	0	1	Post Discharge/Outpati	1	Physical therapy; Occupational therapy;	1	1	1		1	1	0		0	0
C05131	Therapy rehabilitation ICD 9		Basic	0	1	Post Discharge/Outpati	0		0	0	0		0	0	0		0	0
C05132	Therapy or rehabilitation		Basic	0	1	Post Discharge/Outpati	1	1=none; 2=only follow-up, no active treatment;	1	1	1		1	1	0		0	0
C05133	Therapy or rehabilitation		Basic	0	1	Post Discharge/Outpati	1	2-4 weeks; 3 months; 6 months; 12 months; 24	1	1	1		1	1	0		0	0
C05134	Therapy rehabilitation start		Basic	0	1	Post Discharge/Outpati	1	DD-MMM-YYYY	1	1	1		1	1	0		0	0
C05135	Therapy rehabilitation end		Basic	0	1	Post Discharge/Outpati	1	DD-MMM-YYYY	1	1	1		1	1	0		0	0
C05136	Therapy rehabilitation	Yes;No;Unknown;	Basic	0	1	Post Discharge/Outpati	1	Yes;No;Unknown;	1	1	1		1	1	0		0	0
C04814 and 18747	Residence type and other tekst	Hospital;Nursing home;Home;N/A - patient died;Other, specify;Unknown;	Basic	0	1	Outcomes and End Points	1	no; out-patient rehabilitation; general rehab unit; TBI rehabilitation unit; general	1	1	1		1	1	0		0	0
C07442	Satisfaction with Life Scale (SWLS) -	1;2;3;4;5;6;7;	Basic	0	1	Outcomes and End Points	0		0	0	1		1	1	0		0	0
C19546 + C19547 + C07112	Craig Handicap and Assessment Reporting	Less than 10,000; 10,000–15,000; 15,000–20,000;	Basic	0	1	Outcomes and End Points	0		0	0	1		1	1	0		0	0

CDE ID	CENTER-TBI VS TRACK-TBI: In Harmony	CENTER-TBI VS TRACK-TBI: Harmonizable (cfr NIHv2)	CENTER-TBI VS ADAPT: In Harmony	CENTER-TBI VS ADAPT: Harmonizable (cfr NIHv2)	TRACK-TBI VS ADAPT: In Harmony	TRACK-TBI VS ADAPT: Harmonizable (cfr NIHv2)
C00207	1	1	0	0	0	0
C01006	1	1	1	1	1	1
C01005	1	1	1	1	1	1
C01024	0	1	0	1	1	1
C01023	0	1	0	1	1	1
C05130	1	1	0	1	0	1
C05131	0	1	0	1	0	1
C05132	1	1	0	1	0	1
C05133	1	1	0	1	0	1
C05134	1	1	0	1	0	1
C05135	1	1	0	1	0	1
C05136	1	1	0	1	0	1
C04814 and 18747	1	1	0	1	0	1
C07442	0	1	0	0	0	0
C19546 + C19547 + C07112	0	1	0	0	0	0

In comparison with NIH on 20/08/2015							CENTER-TBI Presen		Compatibl	Harmonisation H+P+C	TRACK-TBI Presen		Compatibl	Harmonisation H+P+C	ADAPT Presen		Compatibl	Harmonisation H+P+C
TOTAL:				15			10		10	10	14		14	14	4		4	4
Basic:				15			10		10	10	14		14	14	4		4	4

	CENTER -TBI VS TRACK-TBI: In	CENTER -TBI VS TRACK-TBI: Harmonizable (cfr	CENTER -TBI VS ADAPT: In	CENTER -TBI VS ADAPT: Harmonizable (cfr	TRACK-TBI VS ADAPT: In	TRACK-TBI VS ADAPT: Harmonizable (cfr
TOTAL:	10	15	2	12	4	12
Basic:	10	15	2	12	4	12

Color Scheme of rows	
Dark orange	Excluded – non-global
Light orange	Excluded subpopulation
Green	Merged element
Yellow	Reduced element

General Core CDEs

NIH v2 - GENERAL Core						CENTER-TBI			Harmonisation
CDE ID	CDE Name	CDE Value	Equals TBI Core	Count Core	CDE ID TBI	Present	CDE Value	Compatible	P+C
C00007	Birth date		1	1	C00007	1	age: single field	0	0
C00035	Gender type	Female;Male; Unknown; Unspecified;Not reported;	1	1	C00035	1	male; female; unknown	1	1
C00020	Ethnicity USA category	Hispanic or Latino; Not Hispanic or Latino;Unknown; Not reported;	1	1	C00020	0		0	0
C00030	Race USA category	American Indian or Alaska Native; Asian;Black or African-American; Native Hawaiian or Other Pacific Islander;White; Unknown;Not Reported;	1	1	C00030	0		0	0
C00015	Education year count		1	1	C00015	1	0-30; 99=unknown	1	1
C00322	Medical history condition text	Cardiac Arrhythmia; Cardiomyopathy; Cognitive Impairment, not otherwise specified; Constipation; Contractures; Delayed Puberty; Fractures;Kidney Stones;Mental Retardation (Full scale IQ < 70 documented); Psychiatric Disorder (e.g., attention deficient hyperactivity disorder, anxiety, autism/ autism spectrum, depression, obsessive compulsive disorder);Obesity; Osteoporosis; Pneumonia; Respiratory Insufficiency; Scoliosis;Skin Breakdown;Other, specify;	1	1	C00322	1	Pre-injury ASA-PS Classification system; Medical History Codes (Cardiovascular, Endocrine, Eye Ear Nose Throat, Gastrointestinal, Hematologic, Hepatic, Musculoskeletal, Neurologic, Neurologic, Prev. TBI / concussions, Oncologic, Pulmonary, Psychiatric, Renal, Developmental history, Other); Medication	1	1
C00313	Medical history condition SNOMED CT code		1	1	C00313	0		0	0

CDE ID	TRACK-TBI			Harmonisation	ADAPT			Harmonisation
	Present	CDE Value	Compatible	P+C	Present	CDE Value	Compatible	P+C
C00007	1	age: single field	0	0	1	MM-DD-YYYY	1	1
C00035	1	male; female	1	1	1	male; female	1	1
C00020	0		0	0	1	Hispanic or Latino; Not Hispanic or Latino	1	1
C00030	0		0	0	1	American Indian or Alaska Native; Asian; Black or African American; ative Hawaiian or Other Pacific Islander; White; More than one race; Unknown or not reported	1	1
C00015	1		1	1	1		1	1
C00322	1		1	1	1		1	1
C00313	0		0	0	1		1	1

CDE ID	CENTER-TBI VS TRACK-TBI		CENTER-TBI VS ADAPT		TRACK-TBI VS ADAPT	
	In Harmony	Harmonizable (cfr NIHv2)	In Harmony	Harmonizable (cfr NIHv2)	In Harmony	Harmonizable (cfr NIHv2)
C00007	1	1	1	1	1	1
C00035	1	1	1	1	1	1
C00020	0	0	0	0	0	0
C00030	0	0	0	0	0	0
C00015	0	1	1	1	1	1
C00322	0	1	1	1	1	1
C00313	0	1	0	1	0	1

In comparison with NIH on 20/08/2015									Harmonisation				Harmonisation				Harmonisation	CENTER -TBI VS TRACK-TBI		CENTER -TBI VS ADAPT		TRACK-TBI VS ADAPT	
						Presen		Compatibl	H+P+C	Presen		Compatibl	H+P+C	Presen		Compatibl	H+P+C	In	Harmonizable (cfr	In	Harmonizable (cfr	In	Harmonizable (cfr
TOTAL:				7		4		3	3	4		3	3	7		7	7	2	5	4	5	4	5
Core:				7		4		3	3	4		3	3	7		7	7	2	5	4	5	4	5
Basic:																							

Color Scheme of rows	
Dark orange	Excluded – non-global
Light orange	Excluded subpopulation
Green	Merged element
Yellow	Reduced element
