# Supplementary material for: Common Data Elements: Critical Assessment of Harmonization between Current Multi-Center Traumatic Brain Injury Studies
Source: J Neurotrauma. 2020 May 21;37(11):1283–90. doi: 10.1089/neu.2019.6867 (PMC7249452; doi:10.1089/neu.2019.6867)
Supplement: Supplemental data [file Supp_Tables1-2.pdf]

Supplementary Data

Supplementary Table S1. Unique Common Data Elements per Domain

	Acute, hospitalized		*Rehabilitation for moderate/severe TBI*	
Number	Code	CDE name	Code	CDE name
1.	C05419	Abusive head trauma likelihood type	C00207	Marital or partner status
2.	C05401	Injury date reliability type	C01006	Pupil left eye measurement
3.	C05408	Hospital admission date and time	C01005	Pupil right eye measurement
4.	C00008	Age value	C01024	Pupil shape left eye type
5.	C02505	Marshall CT classification code	C01023	Pupil shape right eye type
6.	C05453	Hypotensive episode indicator	C05130	Therapy or rehabilitation type
7.	C05457	Hypoxic episode indicator	C05131*	Therapy rehabilitation ICD 9 CM code
8.	C05459	Cardiac arrest indicator	C05132	Therapy or rehabilitation frequency
9.	C18674	Emergency room discharge destination type other text	C05133	Therapy or rehabilitation session duration
10.	C04803	Emergency room discharge destination reason	C05134	Therapy rehabilitation start date time
11.	C04807	Vital status	C05135	Therapy rehabilitation end date time
12.	C01011	Glasgow Coma Scale (GCS) - confounders type	C05136	Therapy rehabilitation ongoing indicator
13.			C04814 and C18747	Residence type
14.			C07442	Satisfaction with Life Scale (SWLS) - Life close ideal score
15.			C19546 + C19547 + C07112	Craig Handicap and Assessment Reporting Technique (CHART-SF) - Combined family income indicator

*Element C05131 has been replaced by C21667 ''Therapy rehabilitation ICD 10 CM code'' in the current version of National Institute of Neurological Disorders and Stroke CDEs.

TBI, traumatic brain injury; CDE, Common Data Element; CT, computed tomography; ICD, International Classification of Diseases.

Supplementary Table S2. Retained and Excluded Elements for Comparison with ADAPT

	Excluded from matching process	*Retained elements for matching*
Core	- Employment expanded status	
Basic AH	- Military deployment indicator<br>- Loss of consciousness indicator<br>- Loss of consciousness verification type<br>- Post-traumatic amnesia indicator<br>- Post-traumatic amnesia verify type<br>- Alteration of consciousness indicator<br>- Alteration of consciousness duration range<br>- Alteration of consciousness verify type	- Education school participation<br>- Abusive head trauma<br>- Pediatric GOSE
Basic Rehab	- Marital or partner status<br>- Military deployment indicator<br>- Loss of consciousness indicator<br>- Loss of consciousness verification type<br>- Post-traumatic amnesia indicator<br>- Post-traumatic amnesia verify type<br>- Alteration of consciousness indicator<br>- Alteration of consciousness duration range<br>- Alteration of consciousness verify type<br>- Satisfaction with Life Scale<br>- Craig Handicap and Assessment Scale (CHART-SF)	- Education school participation<br>- Pediatric GOSE

AH, Acute Hospitalized; GOSE, Glasgow Outcome Scale-Extended; CHART-SF, Craig Handicap Assessment Reporting Technique Short Form.
