# Supplementary material for: Common Data Elements: Critical Assessment of Harmonization between Current Multi-Center Traumatic Brain Injury Studies
Source: J Neurotrauma. 2020 May 21;37(11):1283–90. doi: 10.1089/neu.2019.6867 (PMC7249452; doi:10.1089/neu.2019.6867)
Supplement: Supplemental data [file Supp_Tables3-3A.pdf]

Supplementary Table S3. Compatibility of Study Elements Present with CDE Coding

Summary of compatibility with TBI CDEs	*CENTER-TBI*	*TRACK-TBI*	*ADAPT*
Total *N* Compatible elements/unique elements present	69/71 (97%)	73/74 (99%)	56/58 (97%)

TBI, traumatic brain injury; CDEs, Common Data Elements; CENTER-TBI, Collaborative European NeuroTrauma Effectiveness Research in Traumatic Brain Injury; TRACK-TBI, Transforming Research and Clinical Knowledge in Traumatic Brain Injury; ADAPT, Approaches and Decisions in Acute Pediatric Traumatic Brain Injury.

Supplementary Table S3A. Core Elements Not Present/Not Compatible in the Studies

	CENTER-TBI	*TRACK-TBI*	*ADAPT*
Core: Harmonizable elements	21	21	20
CORE – Not present			
General core			
C00007 Birth date	x	x	
C00313 Medical history condition Snomed CT	x	x	
TBI Core			
C17396 Sex participant or subject genotype	x	x	x
C18614 Education primary caregiver-Year count		x	
C18658 Employment Expanded status		x	x
C01053 Loss of consciousness			x
C01055 Post traumatic amnesia range			x
Number Core elements present	18	16	17*
CORE – Not compatible			
General Core	0	0	0
TBI Core			
C05421 Injury ICD external cause code	x		
Number Core elements present and compatible	17	16	17

[#]ADAPT also included C00030 (Race USA category) and C00020 (Ethnicity USA category). However, these elements were excluded as not being globally applicable. The element C18658 Employment Expanded status is not applicable to ADAPT. Corrected for this the compatibility of ADAPT is 17/20 = 85%.

*Supplementary File S1 lists 19 CDEs as present in ADAPT, but this includes the elements C00012 (Education level US type) and C00013 (Education level primary caregiver USA type), which were excluded from analyses.

CENTER-TBI, Collaborative European NeuroTrauma Effectiveness Research in Traumatic Brain Injury; TRACK-TBI, Transforming Research and Clinical Knowledge in Traumatic Brain Injury; ADAPT, Approaches and Decisions in Acute Pediatric Traumatic Brain Injury; Snomed, Systematized Nomenclature of Medicine; CT, computed tomography.
