# Supplementary material for: Common Data Elements: Critical Assessment of Harmonization between Current Multi-Center Traumatic Brain Injury Studies
Source: J Neurotrauma. 2020 May 21;37(11):1283–90. doi: 10.1089/neu.2019.6867 (PMC7249452; doi:10.1089/neu.2019.6867)
Supplement: Supplemental data [file Supp_Table3B.pdf]

Supplementary Table S3B. Basic AH Elements Not Present/Not Compatible in the Studies

	CENTER-TBI	*TRACK-TBI*	*ADAPT*
Number harmonizable AH Basic elements	**46**	**46**	**41**
AH Basic – Not present			
C05433 Protective devices use indicator			x
C05434 Vehicular protective device-type		x	x
C05435 Airbag deploy indicator			x
C00227 Military deployment indicator	x		x[#]
C04803 Emergency room discharge reason		x	x
C01011 Glasgow Coma Scale confounder	x		
C01052 Loss of consciousness indicator			x[#]
C01032 Loss of consciousness verification type			x[#]
C01037 Post-traumatic amnesia indicator			x[#]
C01033 Post-traumatic amnesia verify type			x[#]
C01041 Alteration of consciousness indicator			x[#]
C01045 Alteration of consciousness duration range			x[#]
C01031 Alteration of consciousness verify type			x[#]
C02480 SDH mixed density-CSF like collection indicator	x		
Number AH Basic elements present	**43***	**44***	**37***
Common basic elements	33	34	26
Unique AH basic elements	10	10	11
AH Basic – Not compatible			
C04808 Death cause ICD-9	x	x	x
C02505 Marshall CT Classification			x
Number AH Basic present and compatible	**42**	**43**	**35**
Common basic elements	32	33	25
Unique AH basic elements	10	10	10

[#]These elements ($n = 8$) are not applicable to ADAPT as they are not relevant to the study population of pediatric patients with severe traumatic brain injury. Three basic AH variables that had been excluded from the comparisons for adult studies are relevant to the pediatric population of ADAPT; these concern ''education school participation,'' ''abusive head trauma,'' and the ''pediatric GOS.'' These elements were present in ADAPT. Recalculation of the presence and compatibility of CDEs in ADAPT results in 35/41 (85%).

*Supplementary File S1 lists 46 elements as present for CENTER, but this includes elements C00012 (Education level USA type), C00013 (Education level primary caregiver USA type), and C00202 (Education school participation status), which were excluded from analysis. For TRACK, 45 elements are listed as present in Supplementary File 1, but this includes C18622 (Brief Symptom Inventory), excluded because of not being globally applicable. For ADAPT, Supplementary File 1 lists 41‘ elements as present, but this includes elements C00012 (Education level USA type), C00013 (Education level primary caregiver USA type), excluded as not being globally applicable and C01052 (Loss of consciousness indicator) and C01032 (Loss of consciousness verification type). These elements were not coded in the e-CRF, but loss of consciousness was an inclusion criterion for ADAPT, and thus implicitly present in all subjects.

CENTER-TBI, Collaborative European NeuroTrauma Effectiveness Research in Traumatic Brain Injury; TRACK-TBI, Transforming Research and Clinical Knowledge in Traumatic Brain Injury; ADAPT, Approaches and Decisions in Acute Pediatric Traumatic Brain Injury; AH, Acute Hospitalized; SDH, subdural hematoma; CSF, cerebral spinal fluid; ICD, International Classification of Diseases; CT, computed tomography; GOS, Glasgow Coma Scale, CRF, Case Report Form.
