# Supplementary material for: Common Data Elements: Critical Assessment of Harmonization between Current Multi-Center Traumatic Brain Injury Studies
Source: J Neurotrauma. 2020 May 21;37(11):1283–90. doi: 10.1089/neu.2019.6867 (PMC7249452; doi:10.1089/neu.2019.6867)
Supplement: Supplemental data [file Supp_Table3C.pdf]

Supplementary Table S3C. Basic Rehab Elements Not Present/Not Compatible in the Studies

	CENTER-TBI	*TRACK-TBI*	*ADAPT*
Number Harmonizable Basic rehab elements	**50**	**50**	**41**
Rehab Basic – Not present			
C00207 Marital or partner status			x[#]
C05433 Protective devices use indicator			x
C05434 Vehicular protective device		x	x
C05435 Airbag deploy indicator			x
C00227 Military deployment indicator	x		x[#]
C01024 Pupil shape left eye	x		
C01023 Pupil shape right eye	x		
C01052 Loss of consciousness indicator			x[#]
C01032 Loss of consciousness verification type			x[#]
C01037 Post-traumatic amnesia indicator			x[#]
C01033 Post-traumatic amnesia verification type			x[#]
C01041 Alteration of consciousness indicator			x[#]
C01045 Alteration of consciousness duration range			x[#]
C01031 Alteration of consciousness verify type			x[#]
C02480 SDH mixed density – CSF like collection indicator	x		
C05130 Therapy and Rehab type			x
C05131 Therapy Rehab ICD 9 code	x	x	x
C05132 Therapy or Rehab frequency			x
C05133 Therapy or Rehab session duration			x
C05134 Therapy or Rehab start date/time			x
C05135 Therapy or Rehab end date/time			x
C05136 Therapy or Rehab ongoing indicator			x
C04814 Residence type			x
C07442 Satisfaction with Life Scale (SWLS)	x		x[#]
C19546 Craig Handicap and Assessment Scale (CHART-SF)	x		x[#]
Number Basic Rehab elements present	**43***	**48***	**30***
Common basic elements	33	34	26
Unique Rehab elements	10	14	4
Rehab basic – Not compatible			
C04808 Death cause ICD-9	x	x	x
Number Basic rehab present and compatible	**42**	**47**	**29**
Common Basic elements	32	33	25
Unique Rehab elements	10	14	4

[#]These elements ($n = 11$) are not applicable to ADAPT as they are not relevant to the study population of pediatric patients with severe traumatic brain injury. Two basic Rehab variables that had been excluded from the comparisons for adult studies are relevant to the pediatric population of ADAPT; these concern "education school participation" and the "pediatric GOS." These elements were present in ADAPT. Recalculation of the presence and compatibility of Rehab CDEs in ADAPT results in 29/41 (71%).

*For CENTER, Supplementary File S1 lists 46 elements as present, but this includes but this includes elements C00012 (Education level USA type), C00013 (Education level primary caregiver USA type), and C00202 (Education school participation status), which were excluded from analysis. For TRACK, Supplementary File S1 lists 49 elements as present, but this includes C18622 (Brief Symptom Inventory), excluded because of not being globally applicable. For ADAPT, Supplementary File S1 lists 31 elements as present, but this includes C00013 (Education level primary caregiver USA type), which was excluded from analysis.

CENTER-TBI, Collaborative European NeuroTrauma Effectiveness Research in Traumatic Brain Injury; TRACK-TBI, Transforming Research and Clinical Knowledge in Traumatic Brain Injury; ADAPT, Approaches and Decisions in Acute Pediatric TBI; SDH, subdural hematoma; CSF, cerebral spinal fluid; ICD, International Classification of Diseases.
