# Supplementary material for: Common Data Elements: Critical Assessment of Harmonization between Current Multi-Center Traumatic Brain Injury Studies
Source: J Neurotrauma. 2020 May 21;37(11):1283–90. doi: 10.1089/neu.2019.6867 (PMC7249452; doi:10.1089/neu.2019.6867)
Supplement: Supplemental data [file Supp_Table4.pdf]

Supplementary Table S4. New Core Elements

CDE ID	*CDE Name*	*Definition/description*	*Classification (e.g., Core)*	*CRF Module*
C21117	Subject name	Name of subject/participant.	Core	Test of Everyday Attention for Children (TEA-Ch)
C52231	Interviewer name	The name of the person interviewing the participant/subject.	Core	Clinician-Administered PTSD Scale (CAPS)
C19247	Subject ID	Subject identification ID	Core	Clinician-Administered PTSD Scale (CAPS)
C19500	Assessment performed date	The date of assessment of the examination being performed.	Core	Clinician-Administered PTSD Scale (CAPS)
C52022	Child and Adolescent Scale of Participation (CASP) - Child name	Name of subject/participant, as part of the Child and Adolescent Scale of Participation (CASP).	Core	Child and Adolescent Scale of Participation (CASP)
C52023	Child and Adolescent Scale of Participation (CASP) - Relative name	Name of participant/subject's relative, or the person filling out the scale, as part of the Child and Adolescent Scale of Participation (CASP).	Core	Child and Adolescent Scale of Participation (CASP)
C52024	Child and Adolescent Scale of Participation (CASP) - Relationship text	Text describing your relationship to the participant/subject, as part of the Child and Adolescent Scale of Participation (CASP).	Core	Child and Adolescent Scale of Participation (CASP)
C06005	Data collected date and time	Date (and time, if applicable and known) the data were collected. This may be the date/time a particular examination or procedure was performed.	Core	Child and Adolescent Scale of Participation (CASP)

CDE, Common Data Element; CRF, Case Report Form; PTSTD, post-traumatic stress disorder.
